# Electrostatic Interactions between Complement Regulator CD46(SCR1-2) and Adenovirus Ad11/Ad21 Fiber Protein Knob

**DOI:** 10.1155/2015/967465

**Published:** 2015-08-19

**Authors:** Carl Z. Chen, Ronald D. Gorham, Zied Gaieb, Dimitrios Morikis

**Affiliations:** Department of Bioengineering, University of California, Riverside, CA 92521, USA

## Abstract

Adenoviruses bind to a variety of human cells to cause infection. Both the B2 adenovirus 11 and B1 adenovirus 21 use protein knobs to bind to complement regulator CD46(SCR1-2) in order to gain entry into host cells. In each complex, the two proteins are highly negatively charged but bind to each other at an interface with oppositely charged surface patches. We computationally generated single-alanine mutants of charged residues in the complexes CD46(SCR1-2)-Ad11k and CD46(SCR1-2)-Ad21k. We used electrostatic clustering and Poisson-Boltzmann free energy calculations to propose a hypothesis on the role of electrostatics in association. Our results delineate specific interfacial electrostatic interactions that are critical for association in both CD46(SCR1-2)-Ad11k and CD46(SCR1-2)-Ad21k. These results will serve as a predictive tool in the selection of mutants with desired binding affinity in experimental mutagenesis studies. This study will also serve as a foundation for the design of inhibitors to treat adenovirus infections.

## 1. Introduction

Adenoviruses are viral pathogens that target a variety of organs in the human body [1, 2]. They are highly contagious and can be deadly against patients with a compromised immune system [1, 3–7]. There are over 50 serotypes of adenovirus (Ad), divided among 7 species (A–G) [1, 3–5, 7–15]. Species B viruses are distinguishable from their ability to severely infect the respiratory tract, urinary tract, and kidney. Some subspecies like the B2 adenovirus 11 are primarily responsible for urinary tract infections, while others like the B1 adenovirus 21 are more associated with ocular and respiratory diseases [5, 7–9, 11, 16]. Since no specialized treatments against these B adenoviruses are currently available, it is of particular interest to study how they interact with the immune system at the molecular level.

Species B adenoviruses have broad infectivity tropism by using the ubiquitous CD46 receptor to infiltrate cells [2, 4, 9, 13, 14]. CD46 is a membrane cofactor protein (also known as MCP) that is found as a glycoprotein on all human nucleated cells, including those in the immune system like monocytes and lymphocytes [3, 8, 15, 17–19]. CD46 is a regulator of complement activation (RCA) and belongs to a family of proteins whose structures consist of short consensus repeat (SCR) modules. CD46 works as a cofactor with factor I, a serine protease that cleaves and inactivates complement proteins C3b and C4b. By binding to C3b and C4b, CD46 promotes their degradation [6, 9–12, 14–16, 18]. That is, it works as a suppressing agent of the immune system by preventing attack on autologous cells. Studies have shown that binding of adenovirus, like Ad11, can lead to CD46 downregulation, which sensitizes cells to complement-mediated lysis by MAC [14, 19]. After species B adenoviruses attach to CD46 on a cell, viral invasion of the cell occurs with endocytosis and macropinocytosis, supported by integrins [2, 6].

Due to the ubiquitous nature of CD46, type B adenoviruses have become useful as gene delivery vectors for being able to transduce hematopoietic stem cells, dendritic cells, and malignant tumor cells [2, 3, 7, 8]. Compared with other species, type B adenoviruses are less susceptible to inactivation by host immune molecules due to lower levels of neutralizing antibodies in human sera against the virus [13]. Many recombinant type B or fiber-swapped adenovirus vectors have been developed for gene transfer and vaccination approaches [16]. Thus, the comparison of receptor binding mechanisms to CD46 for different adenoviruses will be useful for improving selection of gene delivery vectors [5, 13].

**Figure 1:**
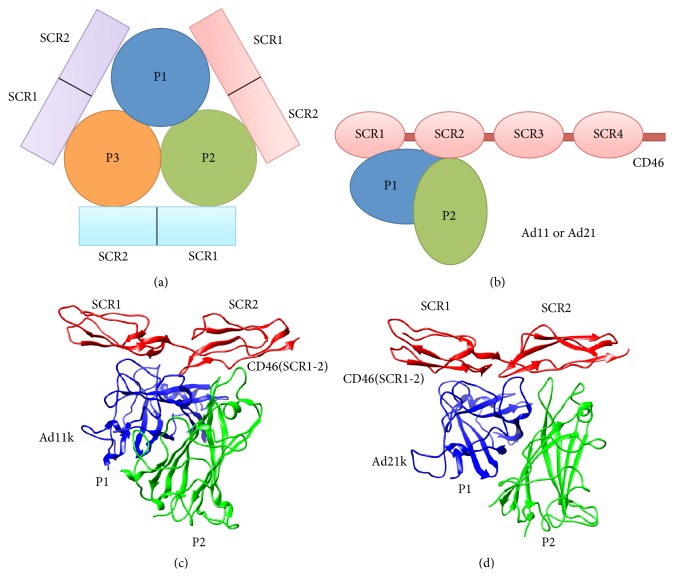
CD46(SCR1-2) and adenovirus protein configuration. (a) Geometric configuration for Ad11k or Ad21k in complex with CD46. Ad11k or Ad21k has three protomers that can bind up to three CD46 molecules, adopting a trimer of dimers conformation. The interactions are homologous for each CD46 molecule. (b) Adenovirus protein binding occurs at the SCR1-SCR2 domains of CD46. Both Ad11k and Ad21k use two protomers, P1 and P2, to bind CD46 at the SCR1 and SCR2 domains. (c) Structure of CD46(SCR1-2)-Ad11k (PDB ID 3O8E). P1 (blue) refers to chain A and P2 (green) refers to chain E of the PDB file. (d) Structure of CD46(SCR1-2)-Ad21k (PDB ID 3L89). P1 (blue) refers to chain B and P2 (green) refers to chain C of the PDB file. In both complexes, P1 buries a greater surface area into CD46(SCR1-2). Molecular graphics were generated using Chimera [28].

Each of the many capsid vertices on adenoviruses 11 and 21 has a trimeric fiber protein consisting of an N-terminus, an elongated shaft, and a globular knob domain that binds with CD46 [1, 2, 6, 7, 11, 19]; the knob domains of Ad11 and Ad21 are called Ad11k and Ad21k, respectively, hereafter. Crystal structures show that the viral knob domain is a trimeric ligand with three identical protomers, and each protomer binds to a specific part of a CD46 molecule (Figure 1(a)). CD46 is made of four SCR domains, with the 4th SCR domain (SCR4) linked by an STP (rich in serine, threonine, and proline) segment, a transmembrane region, and a cytoplasmic tail [1, 5, 6, 8–10, 12, 16–18]. SCR domains are connected to each other by a flexible interdomain linker, and the cytoplasmic tail is attached to the cell surface. Binding of an adenovirus protomer only occurs at the SCR1 and SCR2 domains of CD46(SCR1-2), as shown schematically in Figure 1(b) [2, 5, 7, 8, 17–19].

Crystallization studies show that three protomers (P1, P2, and P3) of Ad11k or Ad21k bind to three molecules of CD46(SCR1-2) [1, 2, 6, 7, 17, 19] (Figure 1(a)). According to the topology of Figures 1(c) and 1(d), each CD46(SCR1-2) molecule binds two protomers of Ad11k or Ad21k. However, one protomer in each complex contacts a larger surface area on CD46(SCR1-2) and is responsible for a larger number of intermolecular interactions.

Both conformation and electrostatics are thought to drive the interaction of the adenovirus ligands to their receptor. The free structure of CD46(SCR1–4) shows a bend between SCR1 and SCR2, but CD46(SCR1-2)-Ad11k and CD46(SCR1-2)-Ad21k show a more linear orientation of these domains, suggesting a conformational change upon virus binding [1, 2, 17]. Also, the loop regions of Ad11k and Ad21k change conformation before binding to CD46(SCR1-2) [1, 2]. Both viral proteins in either complex bind to CD46(SCR1-2) through Coulombic interactions and hydrogen bonds [1, 6]. Previous studies have shown that critical salt bridges exist between Glu63 of CD46(SCR1-2) and specific Arg residues on Ad11k or Ad21k [1, 6, 13–15, 17].

Alanine-scanning mutagenesis has often been used to determine the individual contributions of different residues to a protein of interest [20]. In CD46(SCR1-2)-Ad11k and CD46(SCR1-2)-Ad21k, certain residues can play critical roles in facilitating binding stability. In these residues, the role of the side chain functional groups can be inferred from alanine mutations. However, generating libraries of mutant proteins experimentally can be tedious and time-consuming. Recently, computational alanine scanning has allowed the calculation of alanine mutation effects on the binding free energy of a protein complex [21–25]. The systematic mutation of residues by computational analysis allows the prediction of mutations that can dramatically affect protein binding affinity. Although the methodology is limited by factors such as conformational changes and nonadditive effects, it is an efficient way to yield an energy map of mutational perturbations that reveal important interactions in CD46(SCR1-2)-Ad11k and CD46(SCR1-2)-Ad21k. This energy map can serve as a predictive tool in the selection of CD46(SCR1-2) or adenovirus protein mutants with desired binding affinity in experimental mutagenesis studies.

In this paper, we compare the electrostatic interactions of Ad11k and Ad21k with their CD46(SCR1-2) receptor to delineate similarities and differences in their binding mechanisms. We use computational tools to perturb the electrostatic network of CD46(SCR1-2) and the two adenovirus proteins in order to identify and quantify the role of specific charged amino acids in association and binding. Since CD46(SCR1-2), Ad11k, and Ad21k all have an overall negative charge, long-range recognition of CD46(SCR1-2) with Ad11k/Ad21k is not initially favored. However, if there are localized charged patches on both proteins in a complex, these patches may facilitate long-range recognition as well as specific binding at the interface of the complex. We propose a generalized electrostatic hypothesis that contributes to the formation of the two complexes and suggest specific amino acids that are contributing to binding. Our analysis contributes to a molecular understanding of the mechanisms of adenovirus infection as well as provides a foundation for the design of molecular inhibitors against Ad11k or Ad21k binding.

## 2. Methods

### 2.1. Preparation of Molecular Structures

Three-dimensional coordinates of the complexes CD46(SCR1-2)-Ad11k and CD46(SCR1-2)-Ad21k were obtained from the Protein Data Bank [26], with PDB IDs 3O8E [19] and 3L89 [2], respectively.

For CD46(SCR1-2)-Ad11k, chain A for Ad11k and chain B for CD46(SCR1-2) were selected because they had the least number of missing residues. The missing residues on chain A, 113–128, were terminal residues and were sufficiently away from the binding site to be of concern; therefore, they were not reconstructed. A full heterohexameric CD46(SCR1-2)-Ad11k model was generated by applying a rotation/translation matrix transformation on chains A and B as specified in the PDB file header, using an R (https://www.r-project.org/ [27]) script. The resulting assembly consisted of a total of three CD46(SCR1-2)-Ad11k complexes, according to the topology of Figure 1(c), and a complex containing two protomers bound to a single CD46(SCR1-2) molecule was extracted for our analysis. In this complex, CD46(SCR1-2) contained only modules SCR1 and SCR2, which participate in Ad11k binding, whereas modules SCR3 and SCR4 were deleted. The program Chimera [28] was used to prepare the final CD46(SCR1-2)-Ad11k complex.

For CD46(SCR1-2)-Ad21k, the PDB file had the truncated version of CD46(SCR1-2) with only modules SCR1 and SCR2 present. PDB chains B and C for Ad21k and chain N for CD46(SCR1-2) were chosen for the analysis (Figure 1(d)). Chains B and C were chosen because they had the least missing residues compared to other complexes. The Chimera [28] “model/refine loops” interface of Modeller [29] was used to construct nonterminal missing residues for Ad21k. The missing residues are not located near the complex interface and therefore may not directly affect association.

The modified PDB files for both complexes were used to generate PQR files, containing atomic coordinates, partial charges, and atomic radii using PDB2PQR [30, 31] server with the PARSE [32] force field selection. To determine the ionization state of each ionizable amino acid, apparent p*K*_*a*_ values were precalculated using PROPKA [33, 34] server. This information was used in setting up the Poisson-Boltzmann electrostatic calculations, described below.

### 2.2. Alanine Scanning Mutagenesis

The integrated Analysis of Electrostatic Similarities of Proteins (AESOP) [25] computational framework [23, 24, 35] was used to generate alanine scan mutants of the adenovirus protein, Ad11k or Ad21k, and CD46(SCR1-2) for each of the two complexes. AESOP operates in the R [27] environment using custom-made R scripts to systematically mutate the side chains of charged ionizable residues (Arg, Asp, His, Glu, and Lys) into alanine, one at a time, thus generating a family of mutant proteins. Alanine is typically chosen as a charge-removal perturbation with the least local structural perturbation, in analogy to experimental alanine scan studies. Chimera [28] was used to rename the two adenovirus chains into one chain, enabling the separation of the complex into two components for the electrostatic calculations. For each member of the mutant family, separate PQR files were prepared for the complex and the two individual components of the complex.

### 2.3. Calculation of Electrostatic Potentials

The Adaptive Poisson-Boltzmann Solver (APBS) [36] was used to calculate spatial distributions of electrostatic potentials for the alanine scan mutant family and parent protein, using the linearized Poisson-Boltzmann equation [23, 24] and parameters from the PQR files. The protein molecular surface was calculated using a probe sphere with radius 1.4 Å and the ion accessibility surface was defined using a probe sphere with radius 2.0 Å. Grid dimensions and lengths were determined within AESOP in order to achieve < 1.0 Å grid resolution. For CD46(SCR1-2)-Ad11k, grid dimensions were 161 × 129 × 97 and grid lengths were 135 Å × 96 Å × 116 Å. For CD46(SCR1-2)-Ad21k, grid dimensions were 161 × 129 × 97 and grid lengths were 138 Å × 119 Å × 96 Å. The dielectric coefficient was set to *ε*_*p*_ = 20 for the protein interior and *ε*_*s*_ = 78.54 for the solvent, unless noted otherwise below. The use of an internal dielectric coefficient of *ε*_*p*_ = 20 is justified in a study of the parameterization of the AESOP framework using alanine scan experimental data on protein complexes with available crystal structures [35] and is in agreement with earlier Poisson-Boltzmann parameterization data [37] and numerous studies thereafter. Electrostatic potential calculations were performed with ionic strength corresponding to 150 mM of monovalent counterion concentrations, at a temperature of 298.15 K, to model experimental conditions.

APBS electrostatic potentials were calculated for each of the protein complexes and individual protein components, and electrostatic maps were generated with Chimera [28]. Chimera and custom R scripts were also used for calculation of distances in pairwise electrostatic interactions, such as Coulombic and hydrogen bonding. A cutoff distance of 8 Å was used for Coulombic interactions, with those below 5 Å referred to as salt bridges, hereafter. Chimera was used to calculate hydrogen bonds, according to standard geometric criteria [38]. For each complex, the surface areas of individual proteins and the complex were measured with Chimera, after adding hydrogen atoms to the crystal structures. SASA was then calculated by taking the difference between the sum of individual protein surface areas and the complex surface area. Van der Waals interactions were calculated using distance criteria with custom R scripts, which made use of the CD46(SCR1-2)-Ad11k and CD46(SCR1-2)-Ad21k PDB files.

### 2.4. Calculation of Electrostatic Free Energy of Binding

Six calculations of electrostatic potentials and electrostatic free energies were performed using APBS, as described in [23, 24, 35, 39], with each calculation corresponding to the reactants and products of the thermodynamic cycle shown in Supplementary Figure S1 of the Supplementary Material available online at http://dx.doi.org/10.1155/2015/967465. In preparation for the calculations, all structures of the mutant complexes were centered on the coordinates of the parent protein complex to eliminate grid artifacts and allow for accurate comparison of electrostatic potentials and electrostatic free energies of binding within each family of mutants. After introduction of mutations using AESOP, as described above, the components of each complex were separated to generate the reactants of the thermodynamic cycle of Supplementary Figure S1. The thermodynamic cycle contains a reference binding state (top horizontal process) and a solution binding state (bottom horizontal process). Electrostatic potentials for the solution state were calculated with parameters described above (and shown in Supplementary Figure S1), while those of the reference state were calculated using the same dielectric coefficient of 20 for both the protein interior and solvent, in the absence of ionic strength. Therefore, the vertical processes denote solvation. Electrostatic free energies of binding were calculated for CD46(SCR1-2) and its corresponding adenovirus protein to show mutant effects on thermodynamic stability in each complex. The reported electrostatic free energies of binding for the mutants are relative to the parent protein (also called wild type, WT) and are given as

(1) $$\begin{array}{c} \Delta G^{binding}=\Delta G_{mutant}^{solution}-\Delta G_{parent}^{solution}, \end{array}$$

where Δ*G*_mutant_^solution^ and Δ*G*_parent_^solution^ correspond to

(2) $$\begin{array}{c} \Delta G^{solution}=\Delta \Delta G^{solvation}+\Delta G^{Coulombic} \end{array}$$

as previously described [23, 40] (see Supplementary Figure S1 for description of the electrostatic free energy components).

### 2.5. Electrostatic Clustering of Mutants

Electrostatic similarities for the families of CD46(SCR1-2) and adenovirus protein mutants were calculated using the electrostatic similarity distance (ESD) equation [23, 24, 39]:

(3) $$\begin{array}{c} ESD=\frac{1}{N}\sum_{i,j,k}\frac{\left|\phi_{B}\left(i,j,k\right)-\phi_{A}\left(i,j,k\right)\right|}{\max\left(\left|\phi_{A}\left(i,j,k\right)\right|,\left|\phi_{B}\left(i,j,k\right)\right|\right)}, \end{array}$$

where *φ*_*A*_ and *φ*_*B*_ are the electrostatic potentials of proteins *A* and *B*, respectively, at grid point (*i*, *j*, *k*), and *N* is the total number of grid points. An *n* × *n* pairwise comparison of distance matrix was generated, where *n* is the number of mutants plus the parent protein. An ESD value of 0 represents identical electrostatic potentials, with an increasing value denoting increasing dissimilarity. Mutants were clustered in dendrograms based on their ESD values, using a hierarchical clustering with an average linkage algorithm, implemented in R [23, 24, 39]. Clustering was performed for proteins in each of the two complexes, CD46(SCR1-2)-Ad11k and CD46(SCR1-2)-Ad21k.

## 3. Results

Our goal is to elucidate the role of charge in the interaction of CD46(SCR1-2) with Ad11k and Ad21k. Although CD46(SCR1-2), Ad11k, and Ad21k have negative net charges of −6, −8, and −6, respectively, they are able to form the complexes CD46(SCR1-2)-Ad11k and CD46(SCR1-2)-Ad21k. We used electrostatic potentials, electrostatic free energies of binding, hierarchical clustering, and local physicochemical analysis at the protein-protein interfaces to propose a mechanism of association.

**Figure 2:**
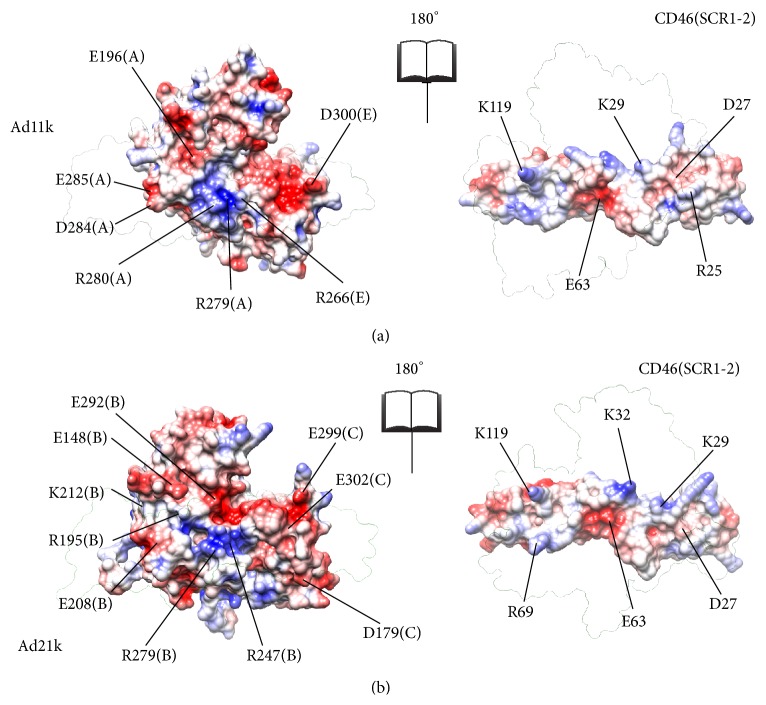
Electrostatic maps of binding sites for CD46(SCR1-2)-Ad11k (a) and CD46(SCR1-2)-Ad21k (b). Both complexes use localized positive and negative patches for complementary binding. Each panel is an open-book view of each complex, where the left image reflects the right image around the vertical axis. The transparent outlines show the position of the partner protein in the complex. Acidic residues reside in red spots (−3*k*_B_T/e) while basic residues reside in blue spots (+3*k*_B_T/e). Residues from Table 1 that contribute to distinguishable electrostatic regions are labeled. Ad11k and Ad21k are composed of two different chains, as indicated by the letter in parentheses. Molecular graphics were generated using Chimera [28].

The surface at the interface of CD46(SCR1-2) with Ad11k/Ad21k is composed of localized regions of positive and negative electrostatic potential projections (Figure 2), which contribute to the makeup of the charged macrodipoles of the proteins, and therefore to nonspecific, long-range recognition step of association. These regions are organized into distinct patches, reflecting the types of residues that dominate the patches, and also contribute to the binding step of association through specific short-range electrostatic interactions. For example, positive patches contain an excess of Arg and Lys residues, while negative patches contain an excess of Asp and Glu residues. Visual inspection of Figure 2 reveals a positive center around Arg280(A) in Ad11k and around Arg247(B)/Arg279(B) in Ad21k with a negative patch on top of it. Complementary patches are in the CD46(SCR1-2) contacts, with a negative center around Glu63 and a positive patch above it.

**Figure 3:**
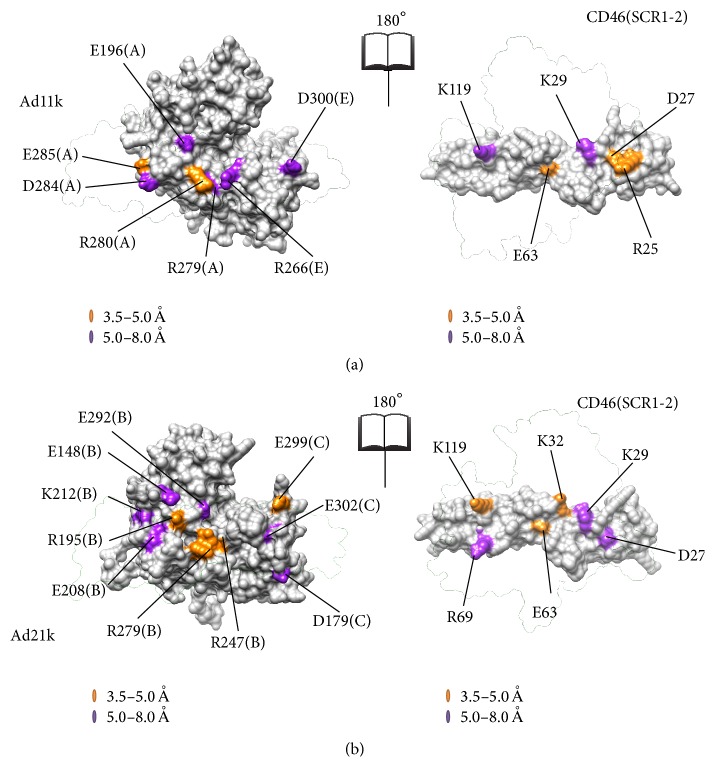
Coulombic interaction maps for CD46(SCR1-2)-Ad11k (a) and CD46(SCR1-2)-Ad21k (b). In accordance with Table 1, both complexes have residues that participate in intermolecular Coulombic interactions at the interface. Residues that form only weak interactions are highlighted in purple, while residues that form at least one medium interaction are highlighted in orange. No strong Coulombic interactions are present. Each panel is an open-book view of each complex, where the left image reflects the right image around the vertical axis. The transparent outlines show the position of the partner protein in the complex. Ad11k and Ad21k residues can come from two different chains, as indicated by the letter in parentheses. Molecular graphics were generated using Chimera [28].

Table 1 summarizes all Coulombic interactions at the interface using an 8 Å cutoff distance. Although both complexes distribute their charged residues at the interface for complementary binding, their charge distributions are distinct. The surface maps of Figure 3 show the location of residues that are involved in any Coulombic interactions. CD46(SCR1-2)-Ad11k has two medium-strong favorable Coulombic interactions at 5 Å or less (called salt bridges herein) and five weaker Coulombic interactions in the range of 5–8 Å (called weak Coulombic interactions herein), whereas CD46(SCR1-2)-Ad21k has three salt bridges and four weak Coulombic interactions (Table 1). Both complexes have unfavorable Coulombic interactions, one strong and one weak for Ad11k and one strong and four weak for Ad21k. Overall, both complexes appear to have similar Coulombic interactions contributing to the binding free energies of the two complexes.

Based on the data of Table 1, a central residue for binding is Glu63 of CD46(SCR1-2), located in the intermodular linker of SCR1-SCR2, because it makes salt bridge contacts with positively charged residues in Ad11k/Ad21k. Therefore Glu63 is a key residue for the stability of CD46(SCR1-2)-Ad11k and CD46(SCR1-2)-Ad21k. Previous studies have also pointed out that Glu63 is an important binding residue for Ad11k [2, 6, 13] or Ad21k [2].

Table 2 shows intermolecular hydrogen bonds for all residues in CD46(SCR1-2) and the adenovirus proteins. CD46(SCR1-2)-Ad11k has 11 hydrogen bonds (involving 8 distinct residues in CD46(SCR1-2) and 7 distinct residues in Ad11k), while CD46(SCR1-2)-Ad21k has 8 hydrogen bonds (involving 8 distinct residues in both CD46(SCR1-2) and Ad21k). Most of the additional hydrogen bonds in CD46(SCR1-2)-Ad11k come from the interactions of the Ad11k protomer 1 with CD46(SCR1-2). Overall, the larger hydrogen bond count contributes to a more favorable enthalpic component of the binding free energy for Ad11k.

At the interface, CD46(SCR1-2)-Ad11k has a buried solvent accessible surface area (SASA) of 1841 Å^2^ while the CD46(SCR1-2)-Ad21k has a buried SASA of 2286 Å^2^, meaning that CD46(SCR1-2)-Ad11k has ~19.5% smaller area of interaction than CD46(SCR1-2)-Ad21k. The larger buried SASA of CD46(SCR1-2)-Ad21k also suggests a larger number of water molecules excluded from the binding interface and released in the bulk solvent, therefore contributing to more favorable entropic effects in the binding free energy. At the same time, this larger SASA implies a larger number of side chains with constrained conformational freedom, contributing to compensatory, less favorable entropic effects. We also calculated van der Waals interactions (atom-atom contacts within 4 Å) for each complex and found that CD46(SCR1-2)-Ad11k has 131 (28 from nonpolar atom pairs) interactions, whereas CD46(SCR1-2)-Ad21k has 184 interactions (79 from nonpolar atom pairs), corresponding to 51 more nonpolar contacts for Ad21k compared to Ad11k. This is not unexpected given that CD46(SCR1-2)-Ad21k has higher buried SASA. These van der Waals interactions correspond mainly to hydrophobic contributions to binding. Overall, the larger number of nonpolar interactions contributes to a more favorable enthalpic component of the binding free energy for Ad21k.

Our hydrogen bonding, salt bridge, and SASA data are overall in agreement with data from the PDBePISA server (http://www.ebi.ac.uk/pdbe/prot_int/pistart.html) [41], with the exception of some potentially very weak hydrogen bonds suggested by PDBePISA, corresponding to donor-acceptor distances > 3.5 Å. It should be noted that our analysis includes both favorable and unfavorable Coulombic interactions up to 8 Å. The PDBePISA analysis showed very small but similar gain in the solvation free energy upon formation of both complexes (without including interfacial hydrogen bond and salt bridge contributions).

**Figure 4:**
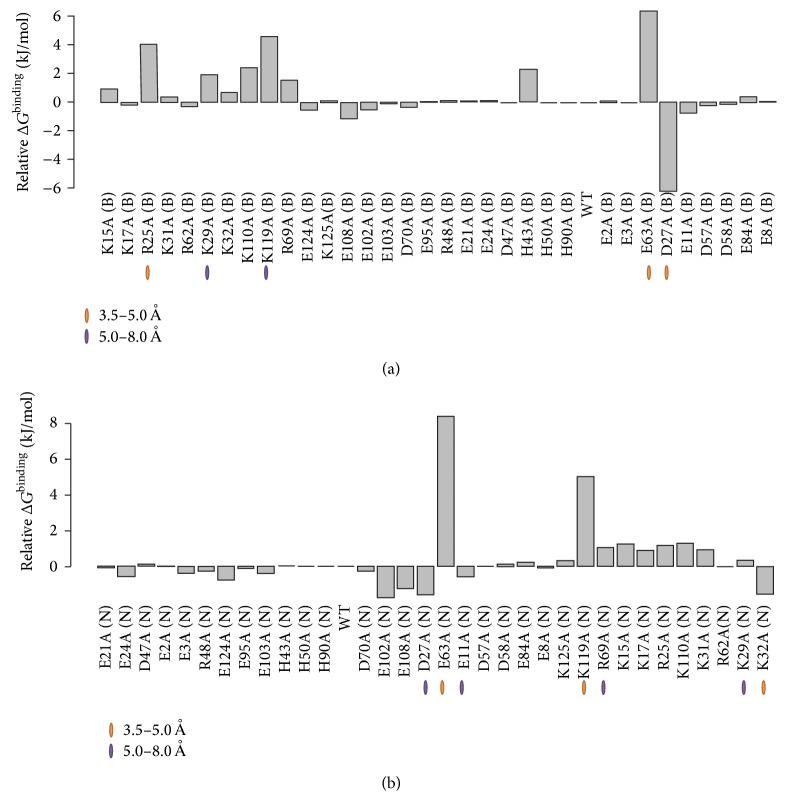
Electrostatic free energies of binding for CD46(SCR1-2) mutants in complex with Ad11k (a) and Ad21k (b). Charged residues in CD46(SCR1-2) were systematically mutated into alanine, one at a time as described in the text, to generate a family of as many mutants as ionizable residues in the protein. The type of residue, residue number, and chain letter (in parentheses) for each mutant are shown. Negative and positive Δ*G* values represent increased and decreased thermodynamic stability of mutant complexes relative to WT (parent), respectively. Highlighted residues are involved in specific binding and are grouped based on the distance of their closest Coulombic interaction. All other charged residues have weak or diminishing Coulombic interactions at more than 8 Å apart. The order of CD46(SCR1-2) mutants in (a) and (b) corresponds to the order in the dendrograms of Figures S4 and S5, respectively.

**Figure 5:**
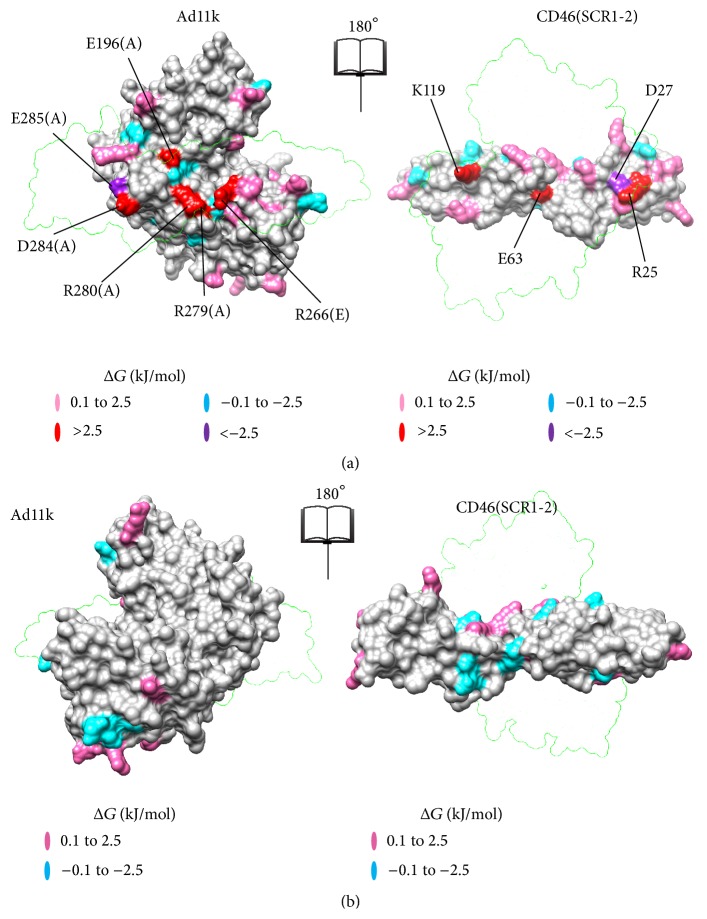
Maps of electrostatic free energy of binding for CD46(SCR1-2)-Ad11k at the binding site (a) and at the surface opposite to the binding site (b). The maps show the effect of mutating individual charged residues on Δ*G* ^binding^ for the complex. Loss of binding mutants is shown in pink (low; Δ*G* ^binding^ = 0.1 to 2.5 kJ/mol, where 2.5 kJ/mol is the thermal energy at room temperature) or red (high; Δ*G* ^binding^ > 2.5 kJ/mol). Gain of binding mutants is shown in cyan (low; Δ*G* ^binding^ = −0.1 to −2.5 kJ/mol) or purple (high; Δ*G* ^binding^ < −2.5 kJ/mol). Other mutants with −0.1 < Δ*G* ^binding^ < 0.1 kJ/mol are grayed out. See Supplementary Material for details. Mutants with high Δ*G* ^binding^ are labeled. Each panel is an open-book view of the complex, where the left image reflects the right image around the vertical axis. (a) and (b) are related by a 180°-rotation about a vertical axis. The transparent outlines show the position of the partner protein in the complex. Molecular graphics were generated using Chimera [28].

**Figure 6:**
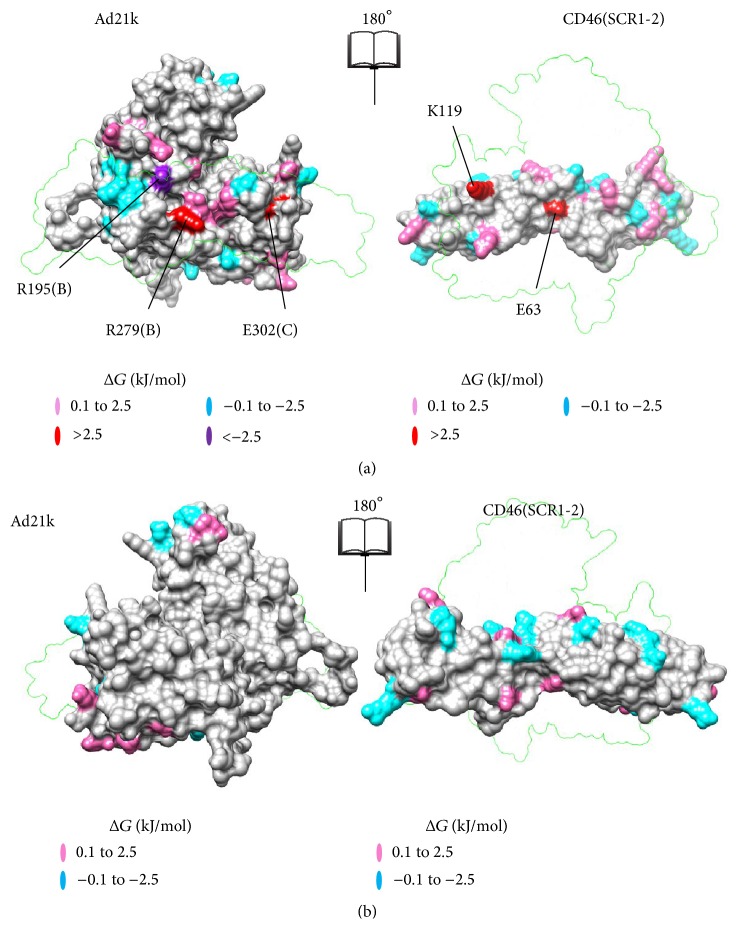
Maps of electrostatic free energy of binding for CD46(SCR1-2)-Ad21k at the binding site (a) and at the surface opposite to the binding site (b). Presentation and notation is as in Figure 5.

Through a systematic alanine scan of charged residues, we determined the contribution of each charged amino acid to the electrostatic component of the binding free energy in both complexes. We calculated electrostatic free energies of binding for the two families of mutants and parent proteins, as described in Section 2.4.  Figure 4 shows graphs of differences between electrostatic free energies of binding (1) for CD46(SCR1-2) from its complexes with the Ad11k (Figure 4(a)) and Ad21k (Figure 4(b)), respectively. Supplementary Figures S2 and S3 show similar graphs for Ad11k and Ad21k. Positive Δ*G* ^binding^ values indicate that the complex is thermodynamically less stable than the parent protein complex, whereas negative Δ*G* ^binding^ values indicate a more stable complex than the parent complex. Thus, a positive Δ*G* ^binding^ sign indicates a loss of binding, whereas a negative Δ*G* ^binding^ sign indicates a gain of binding upon mutation. The free energy maps of Figures 5 and 6 show the location of individual residues that have experienced significant loss or gain of binding (See Supplementary Tables 1–3 for Δ*G* ^binding^ values). In both complexes, the majority of mutations resulted in a loss of binding. Mutated residues that contribute to more than 2.5 kJ/mol in the free energy differences are considered to be more influential for binding, and they are marked in Figure 4 (the chosen 2.5 kJ/mol value corresponds to the thermal energy at room temperature).

The data of Figure 4 quantify that Glu63 of CD46(SCR1-2) has the strongest contribution to binding, followed by Lys119 in both complexes, and also Arg25 in the case of Ad11k. This is in agreement with the assessment of intermolecular interactions discussed above on the basis of visualization and charge-charge distances in Coulombic interactions (Figures 2 and 3). The electrostatic potential of the Glu63Ala mutant appears to be unique, as it clusters on its own in the electrostatic clustering dendrograms (Supplementary Figures S4 and S5), and it shows the largest loss of binding effect in the free energy plots (Figure 4 and Supplementary Table S1), compared to other acidic mutants. It appears that Glu63 is a key residue for the interaction of CD46(SCR1-2) with the adenovirus proteins, perhaps because of its unique position in the intermodule SCR1-SCR2 linker. In the case of the complex of CD46(SCR1-2) with Ad11k, removal of the unfavorable Asp27-Glu285(A) interaction, by replacing Asp27 with Ala, resulted in the strongest predicted gain of binding (Figure 4). In general, the electrostatic free energy data (Figure 4) are in agreement with the distance-based evaluation of Coulombic interactions (Table 1). Similar arguments can be made for the Δ*G* ^binding^ of Ad11k and Ad21k, shown in Supplementary Figures S2 and S3. However, the electrostatic free energy data reveal less obvious weak binding contributions from residues that are beyond the 8 Å distance that was used to define the binding interface. For completion, electrostatic clustering dendrograms for Ad11k and Ad21k are shown in Supplementary Figures S6 and S7.

Although the presence of surface patches with like charges produce unfavorable Coulombic interactions, this effect can be compensated by the introduction of favorable intermolecular Coulombic interactions at the binding interface and by solvation effects, depending on number, magnitude, and location of intermolecular interactions. To delineate the relative Coulombic and solvation contributions in each mutant and parent protein, we examined the raw electrostatic free energies of association, Δ*G* ^solution^, for each mutant and parent protein and their decomposition into Coulombic and solvation contributions, according to the thermodynamic cycle of Supplementary Figure S1. Supplementary Figures S8–S11 show the electrostatic free energy decomposition for each of the four proteins in the CD46(SCR1-2)-Ad11k and CD46(SCR1-2)-Ad21k complexes. As expected Δ*G* ^Coulombic^ and ΔΔ*G* ^solvation^ show opposite trends [35], and their combined magnitudes determine if the much smaller magnitude Δ*G* ^solution^ will be overall favorable (negative) or unfavorable (positive). Based on the values and sign of the calculated Δ*G* ^solution^, CD46(SCR1-2)-Ad11k appears to be electrostatically more stable than CD46(SCR1-2)-Ad21k; however, Δ*G* ^solution^ (Coulombic and solvation effects) may not be the dominant contribution to the overall stability of the complexes, and one should consider additional energetic contributions (hydrogen bonds and nonpolar interactions), as well as entropic contributions.

## 4. Discussion

### 4.1. Complementary Surface Patches Stabilize Both Complexes

**Figure 7:**
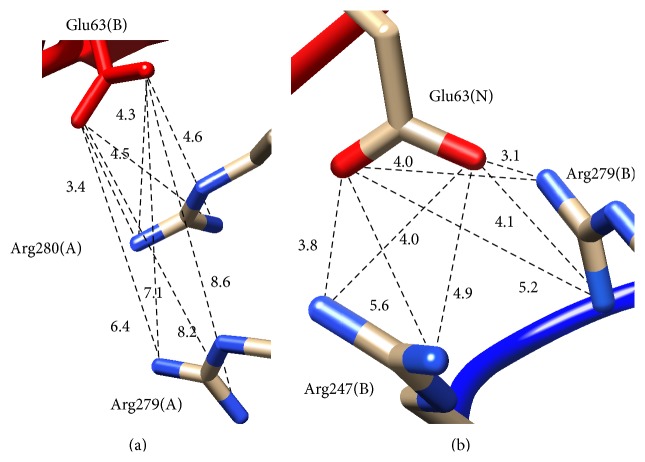
Distances for salt bridge interactions of Glu63 in CD46(SCR1-2) with Arg residues in Ad11k (a) and Ad21k (b). It is noted that, in Ad21k, Glu63 forms a bifurcated salt bridge with Arg247(B) and Arg279(B), whereas, in Ad11k, Glu63 forms a single salt bridge with Arg280(A), using a cutoff distance of 5 Å for salt bridge formation. Molecular graphics were generated using Chimera [28].

The effects of mutations of residues at the complementary charged surface patches discussed above are depicted in the free energy graphs of Figure 4 and are summarized in Supplementary Tables S1–S3. For example, in the case of the CD46(SCR1-2) free energy graph, the Glu63 mutant has a significantly higher (loss of binding) Δ*G* ^binding^ (>6 kJ/mol) than other mutants (Figure 4). The higher Δ*G* ^binding^ for Glu63 comes from its primary contribution to the central negative patch of CD46(SCR1-2), which aligns with the central positive patch of Ad11k and Ad21 upon complex formation (Figure 2). Specifically, Glu63 forms a salt bridge with Arg280(A) in Ad11k and a bifurcated salt bridge with Arg247(B) and Arg279(B) in Ad21k, as shown in Figure 7. Therefore, removing Glu63 would significantly reduce patch complementation and loss of pairwise interactions with the aforementioned Arg residues in Ad11k/Ad21k, with the effect being more prominent in Ad21k because of the bifurcated salt bridge (Table 1, Figure 7).

Unlike the Arg residues of Ad11k/Ad21k, Glu63 does not have nearby negative residues that can compensate for its mutation, so its Δ*G* ^binding^ is considerably higher than other mutations. Thus, there are specific charged residues in the center of Ad11k or Ad21k and CD46(SCR1-2) that destabilize the complex when mutated. Since these residues exist in oppositely charged surface patches, patch complementation and specific pairwise interactions play a significant role in stabilizing both complexes.

Similarly, in the case of the Ad11k free energy graph, the Arg266(E)Ala, Arg279(A)Ala, and Arg280(A)Ala mutants all have relatively higher positive Δ*G* ^binding^ (>3 kJ/mol) than that of the wild type (Supplementary Figure S2). The three residues belong to the same Ad11k central positive patch, which aligns with a central negative patch containing Glu63 on CD46(SCR1-2) (Figure 2). For the Ad21k free energy graph, the Arg247(B)Ala and Arg279(B)Ala mutants also show positive Δ*G* ^binding^ (Supplementary Figure S3) and reside in a positive patch that aligns with Glu63 of CD46(SCR1-2) (Figure 2). Because mutating any of these Arg residues diminishes the corresponding patch size, the higher Δ*G* ^binding^ likely comes from a weakening of patch complementation.

With the distribution of positive and negative charge on both proteins, the patch charge roles can be reversed. For Ad11k, the large negative patch near Asp300(E) complements with the protruded positive patch containing Lys119 on CD46(SCR1-2) (Figure 2). For Ad21k, Glu299(C) also resides in a large negative patch that aligns with Lys119. The Asp300(E)Ala mutant has a Δ*G* ^binding^ of 1.46 kJ/mol and the Glu299(C)Ala mutant has a Δ*G* ^binding^ of 1.22 kJ/mol. These mutations diminish the corresponding negative patch, which would then be less amenable to align with positive patch of Lys119. Granted, there are nearby negative residues that can compensate for the loss of Asp300(E) or Glu299(C), but their absence still results in a less stable complex.

For the Lys119Ala mutant of CD46(SCR1-2), the free energy graph of CD46(SCR1-2) shows a more pronounced Δ*G* ^binding^ of approximately 5 kJ/mol in both complexes (Figure 4). Unlike Asp300(E) of Ad11k or Glu299(C) of Ad21k, Lys119 is responsible for a much more confined charged area. Hence, the mutation of Lys119 would strongly diminish the corresponding positive patch on CD46(SCR1-2). Without Lys119, the large opposing negative patch containing Asp300(E) on Ad11k or Glu299(C) on Ad21k would be heavily compromised in electrostatic alignment. In other words, the high Δ*G* ^binding^ values of Lys119 show how effectively this mutation destabilizes both complexes. For both CD46(SCR1-2)-Ad11k and CD46(SCR1-2)-Ad21k, free energies are a strong indicator that complementary surface patches provide electrostatic stability.

The role of additional complementary mutations involved in stabilizing or destabilizing pairwise interactions, but not located in the charged surface patches discussed above, can be depicted by examining Figure 4, Supplementary Figures S2 and S3, and Supplementary Tables S1–S3. The specific intermolecular Coulombic and hydrogen bonding interactions can be seen in Tables 1 and 2, respectively.

### 4.2. CD46(SCR1-2)-Ad11k Is Stabilized by a Larger Number of Hydrogen Bonds Compared to CD46(SCR1-2)-Ad21k

Cupelli et al. [2] point out that both protein complexes have similar association rate constants (*k*_*a*_) and likely form complexes using similar recognition processes. As Figure 2 shows, this recognition occurs from patch complementation between CD46(SCR1-2) and the adenovirus proteins. However, CD46(SCR1-2)-Ad21k has a much greater dissociation rate constant (*k*_*d*_), resulting in lower binding affinity (higher dissociation constant, *K*_*D*_ = *k*_*d*_/*k*_*a*_). According to Table 2, CD46(SCR1-2)-Ad11k has 43% more intermolecular hydrogen bonds but 19.5% less SASA than CD46(SCR1-2)-Ad21k. We speculate that the additional hydrogen bonds play a dominant role in stabilizing the CD46(SCR1-2)-Ad11k. Nonetheless, the larger binding area of Ad21k would contribute to the stability of the interface because of additional nonpolar interactions. The 22-fold higher affinity of Ad11k compared to Ad21k, expressed as ratio of *K*_*D*_ values, corresponds to ~8 kJ/mol binding free energy difference. Considering that a typical hydrogen bond energy can be up to 6 kJ/mol [42], the 11 hydrogen bonds of CD46(SCR1-2)-Ad11k would contribute up to 66 kJ/mol to the binding affinity, and the 8 hydrogen bonds of CD46(SCR1-2)-Ad21k would contribute up to 48 kJ/mol. The difference of 18 kJ/mol in hydrogen bonding is in favor of CD46(SCR1-2)-Ad11k stabilization compared to CD46(SCR1-2)-Ad21k (note that these contributions are estimates because we do not use variable distance and angle criteria and are used simply to denote which complex may be generally more favorable based on counting hydrogen bonds). Based on qualitative distance arguments, and although we have not taken into account effects owed to position-dependent variable dielectric coefficients, a coarse estimate of Coulombic interactions suggests that Ad11k and Ad21k should have similar electrostatic contributions to binding (*vide supra*). On the other hand, the increased interfacial SASA of CD46(SCR1-2)-Ad21k provides a 9.3 kJ/mol advantage for this complex (using a nonpolar energy approximation as in MM-GBSA calculations [20], with surface tension parameter equal to 0.0209 kJ/mol/Å^2^). In combination, these estimates suggest that CD46(SCR1-2)-Ad11k may be favored energetically over CD46(SCR1-2)-Ad21k, by ~9 kJ/mol. It should be noted that these calculations provide a very approximate estimate of intermolecular energies and neglect the fact that intermolecular contacts may be dynamically forming and deforming. Also, the calculations neglect the frequency of contacts given that we use static crystallographic structures, as well as any entropic effects upon binding and desolvation.

### 4.3. Association Model for CD46(SCR1-2)-Ad11k and CD46(SCR1-2)-Ad21k

The hypothesis underlying our study assumes a two-step electrostatics-based model of association [23–25, 35, 40, 43] between CD46(SCR1-2) and the adenovirus proteins. According to this model, the first step, which is the recognition step, involves nonspecific long-range electrostatic interactions between the two proteins through the interaction of protein macrodipoles. In the second step, which is the binding step, the two proteins undergo conformational and entropic changes to form specific pairwise physicochemical interactions, including hydrophobic and electrostatic (hydrogen bonding and Coulombic/solvation) contacts, as well as solvent exclusion. Although both components of the complexes, CD46(SCR1-2) and Ad11k/Ad21k, have a negative net charge, the presence of complementary surface patches of opposite charges (and electrostatic potentials generated by these charges) is sufficient to accelerate their encounter through long-range electrostatic interactions and to lead to complex formation through short-range pairwise interactions and entropic effects. The presence of patches with like charges can be viewed as a stressed Coulombic environment in the individual proteins, which drives complex formation in order to at least partially alleviate the stress by the formation of intermolecular electrostatic contacts. Therefore, the two-step association model, consisting of weak and nonspecific electrostatic recognition (step 1) and strong and specific binding (step 2), is expected to be operative in the case of CD46(SCR1-2)-Ad11k/Ad21k association. However, in the case of step 2, compensatory enthalpic and entropic contributions may make qualitative arguments less intuitive.

### 4.4. Gain or Loss of Binding Can Be Predicted by the Effect of Computational Alanine Scans on Electrostatic Potentials

Experimental alanine scans (and point mutations in general) are perturbation methods that alter the physicochemical composition of the local environment and are typically used to elucidate the role of the mutated amino acids in binding. Computational alanine scans (or other mutations) and electrostatic calculations are useful to predict the role of ionizable amino acids in binding for highly charged proteins. Such data are faster to generate than experimental data and can guide subsequent experimental studies. Figures 4–6, Supplementary Figures S2 and S3, and Supplementary Tables S1–S3 provide databases of computationally predicted loss of binding and gain of binding mutations for CD46(SCR1-2), Ad11k, and Ad21k.

We have compared our computational results with an experimental alanine scan that examined the interaction of CD46(SCR1-2) mutants with Ad11k [5]. This study suggests that mutating Arg25, Lys110, and Lys119 on CD46(SCR1-2) results in ~20% drop in binding efficiency compared to wild type CD46, which is in agreement with our study. However, the experimental study does not present data for the most significant mutation according to our computational data, that of Glu63Ala. It is likely that Glu63Ala was excluded in the experimental study because it is located in the linker between the two SCR domains; however, our experience with other SCR-containing viral inhibitors of the complement system suggests that charged linker residues play an important role in recognition of complement protein targets [43–46]. The experimental data shows no change in binding efficiency for the Asp27Ala mutant, even though our computational analysis predicts that the mutant should experience a noticeable gain in binding. This may be either due to the experimental design or due to a nonphysiological conformation of Asp27 in the crystal structure of CD46(SCR1-2)-Ad11k, but not in CD46(SCR1-2)-Ad21k, we used in our calculations. To the best of our knowledge, there are no mutagenesis data on CD46(SCR1-2)-Ad21k in the literature. Thus, we hope that our computational results can act as a guide for future mutagenesis experiments in order to target more relevant residues that impact binding on CD46(SCR1-2) and Ad11k/Ad21k.

## 5. Conclusions

Despite the fact that CD46(SCR1-2) and the adenovirus proteins Ad11k/Ad21k have negative net charge, they are still able to associate with stable complexes. This is because of the presence of oppositely charged patches on the surfaces of CD46(SCR1-2) and Ad11k/Ad21k, which allow the adenovirus proteins to recognize CD46(SCR1-2) and promote binding, in addition to nonelectrostatic effects and entropic contributions. Such patches originate from the clustering of specific charged groups on the protein surfaces. At the interface, residues that contribute to binding participate in important pairwise intermolecular interactions (hydrophobic and electrostatic). Our electrostatic free energy graphs show that mutations of ionizable residues at the complex interface cause a more distinguishable change in the electrostatic energy of binding for the complex. A critical residue for the formation of CD46(SCR1-2)-Ad11k/Ad21k is Glu63 of CD46(SCR1-2), which is involved in stabilizing Coulombic interactions in both complexes.

Overall, this study has shown that electrostatics contributes to the formation of CD46(SCR1-2)-Ad11k and CD46(SCR1-2)-Ad21k. We propose specific mutations that are predicted to be stabilizing or destabilizing of the complexes. Upon experimental verification, this type of analysis may be useful in designing CD46(SCR1-2)-like molecules or CD46(SCR1-2)-derived peptide fragments that may be potential Ad11k/Ad21k viral inhibitors. Alternatively, this type of analysis may be useful in designing Ad11k/Ad21k-like proteins or Ad11k/Ad21k-derived peptide fragments to serve as inhibitors of CD46(SCR1-2) and complement activation, which would be useful in cases of autoimmune diseases. Finally, site-specific mutations could be used for developing adenoviruses into better gene delivery vehicles, and combinatorial mutagenesis may lead to tailored protein variants, as previously shown [43–46].

## Supplementary Material

The Supplementary Material contain three Tables with ΔG^binding^ values, and 11 Figures with the thermodynamic cycle used in the calculation of electrostatic free energies of binding, electrostatic free energies of binding, clustering dendrograms of ESDs, and decompositions of electrostatic free energies of binding to ΔG^Coulombic^ , ΔΔG^solvation^ , and ΔG^solution^ .

## Figures and Tables

**Table 1:** Intermolecular Coulombic interactions.^a^

Interactions for CD46(SCR1-2)-Ad11k (chain A)						
CD46(SCR1-2)	Residue number	Atom	Ad11k	Residue number	Atom	Distance (Å)
**Arg**	**25**	**CZ**	**Glu**	**285**	**CD**	**5.0**
Arg	25	CZ	Asp	284	CG	6.3
**A** **s** **p** ^*∗*^	**27**	**CG**	**Glu**	**285**	**CD**	**4.9**
Asp^*∗*^	27	CG	Asp	284	CG	6.6
Lys	29	NZ	Glu	196	CD	7.4
**Glu**	**63**	**CD**	**Arg**	**280**	**CZ**	**4.0**
Glu	63	CD	Arg	279	CZ	7.7

Interactions for CD46(SCR1-2)-Ad11k (chain E)						
CD46(SCR1-2)	Residue number	Atom	Ad11k	Residue number	Atom	Distance (Å)

Glu	63	CD	Arg	266	CZ	7.3
Lys	119	NZ	Asp	300	CG	6.1

Interactions for CD46(SCR1-2)-Ad21k (chain B)						
CD46(SCR1-2)	Residue number	Atom	Ad21k	Residue number	Atom	Distance (Å)

Glu^*∗*^	11	CD	Glu	148	CD	7.7
Asp^*∗*^	27	CG	Glu	208	CD	7.8
Lys^*∗*^	29	NZ	Arg	195	CZ	7.7
Lys^*∗*^	29	NZ	Lys	212	NZ	7.8
**L** **y** **s** ^*∗*^	**32**	**NZ**	**Arg**	**195**	**CZ**	**4.3**
Lys	32	NZ	Glu	148	CD	7.1
Lys	32	NZ	Glu	292	CD	7.9
**Glu**	**63**	**CD**	**Arg**	**247**	**CZ**	**4.5**
**Glu**	**63**	**CD**	**Arg**	**279**	**CZ**	**4.0**

Interactions for CD46(SCR1-2)-Ad21k (chain C)						
CD46(SCR1-2)	Residue number	Atom	Ad21k	Residue number	Atom	Distance (Å)

Arg	69	CZ	Asp	179	CG	6.1
**Lys**	**119**	**NZ**	**Glu**	**299**	**CD**	**4.6**
Lys	119	NZ	Glu	302	CD	7.3

^a^Pairs of residues in bold faced characters denote interactions within 5 Å.

*∗* denotes unfavorable interactions.

**Table 2:** Intermolecular hydrogen bonds.

Interactions for CD46(SCR1-2)-Ad11k (chain A)						
CD46(SCR1-2)	Residue number	Atom	Ad11k	Residue number	Atom	Distance (Å)
Tyr	28	O	Asn	283	ND2	3.7
Cys	30	N	Ile	282	O	3.0
Tyr	36	N	Ala	281	O	2.9
Tyr	36	O	Asn	245	ND2	3.3
Thr	42	N	Asp	284	OD2	2.8
Thr	42	OG1	Asp	284	N	3.1
His	43	ND1	Asp	284	OD2	2.8
Ser	112	OG	Glu	196	OE2	3.1

Interactions for CD46(SCR1-2)-Ad11k (chain E)						
CD46(SCR1-2)	Residue number	Atom	Ad11k	Residue number	Atom	Distance (Å)

Thr	64	O	Arg	266	NH1	2.9
Thr	64	O	Arg	266	NH2	3.5
Ala	114	O	Arg	266	NH1	2.8

Interactions for CD46(SCR1-2)-Ad21k (chain B)						
CD46(SCR1-2)	Residue number	Atom	Ad21k	Residue number	Atom	Distance (Å)

Cys	30	N	Ile	281	O	3.2
Tyr	36	N	Thr	280	O	2.7
Thr	64	OG1	Thr	245	O	2.6
Ser	112	O	Arg	247	NH1	3.3

Interactions for CD46(SCR1-2)-Ad21k (chain C)						
CD46(SCR1-2)	Residue number	Atom	Ad21k	Residue number	Atom	Distance (Å)

Arg	69	O	Asn	304	ND2	3.5
Trp	116	N	Tyr	263	O	3.2
Lys	119	NZ	Ser	300	O	3.0
Pro	120	O	Ser	303	OG	3.3

## Abbreviations

CD46: Cluster of differentiation 46
MCP: Membrane cofactor protein (an alternative name for CD46)
Ad11k: Adenovirus 11 knob domain
Ad21k: Adenovirus 21 knob domain
SCR: Short consensus repeat module
CD46(SCR1-2): CD46 fragment containing SCR1 and SCR2 modules
MAC: Membrane attack complex
ESD: Electrostatic similarity distance.
