# Supplementary material for: Electrostatic Interactions between Complement Regulator CD46(SCR1-2) and Adenovirus Ad11/Ad21 Fiber Protein Knob
Source: Mol Biol Int. 2015 Aug 19;2015:967465. doi: 10.1155/2015/967465 (PMC4556874; doi:10.1155/2015/967465)
Supplement: Supplementary file 1 — The Supplementary Material contain three Tables with ΔGbinding values, and 11 Figures with the thermodynamic cycle used in the calculation of electrostatic free energies of binding, electrostatic free energies of binding, clustering dendrograms of ESDs, and decompositions of electrostatic free energies of binding to ΔGCoulombic , ΔΔGsolvation , and ΔGsolution . [file 967465.f1.pdf]

Supplementary Materials

Tables

Table S1A. CD46(SCR1-2) $\Delta G^{binding}$ values for CD46(SCR1-2)-Ad11k. Bold faced residues are involved in Coulombic interactions. Letters in parentheses show the protein chain where the mutation occurs. Blue color indicates lysine and arginine mutations, and red color indicates aspartic and glutamic acid mutations.

Mutant	$\Delta G^{binding}$ (kJ/mol)	Mutant	$\Delta G^{binding}$ (kJ/mol)
WT	0	**D27A (B)**	**-6.20**
		D47A (B)	-0.03
H43A (B)	2.30	D57A (B)	-0.21
H50A (B)	-0.01	D58A (B)	-0.13
H90A (B)	0.01	D70A (B)	-0.35
K15A (B)	0.92	E2A (B)	0.09
K17A (B)	-0.17	E3A (B)	-0.05
**K29A (B)**	**1.93**	E8A (B)	0.06
K31A (B)	0.40	E11A (B)	-0.74
K32A (B)	0.69	E21A (B)	0.10
K110A (B)	2.43	E24A (B)	0.14
**K119A (B)**	**4.58**	**E63A (B)**	**6.39**
K125A (B)	0.12	E84A (B)	0.40
		E95A (B)	0.06
**R25A (B)**	**4.06**	E102A (B)	-0.50
R48A (B)	0.13	E103A (B)	-0.08
R62A (B)	-0.29	E108A (B)	-1.14
R69A (B)	1.55	E124A (B)	-0.54

Table S1B. CD46(SCR1-2) $\Delta G^{binding}$ values for CD46(SCR1-2)-Ad21k. Bold faced residues are involved in Coulombic interactions. Letters in parentheses show the protein chain where the mutation occurs. Blue color indicates lysine and arginine mutations, and red color indicates aspartic and glutamic acid mutations.

Mutant	$\Delta G^{binding}$ (kJ/mol)	Mutant	$\Delta G^{binding}$ (kJ/mol)
WT	0	**D27A (N)**	**-1.61**
		D47A (N)	0.12
H43A (N)	0.05	D57A (N)	-0.01
H50A (N)	0.02	D58A (N)	0.11
H90A (N)	0	D70A (N)	-0.30
K15A (N)	1.25	E2A (N)	-0.01
K17A (N)	0.90	E3A (N)	-0.41
**K29A (N)**	**0.34**	E8A (N)	-0.10
K31A (N)	0.94	**E11A (N)**	**-0.61**
**K32A (N)**	**-1.59**	E21A (N)	-0.09
K110A (N)	1.31	E24A (N)	-0.58
**K119A (N)**	**5.04**	**E63A (N)**	**8.44**
K125A (N)	0.31	E84A (N)	0.24
		E95A (N)	-0.12
R25A (N)	1.16	E102A (N)	-1.76
R48A (N)	-0.28	E103A (N)	-0.39
R62A (N)	-0.02	E108A (N)	-1.26
**R69A (N)**	**1.06**	E124A (N)	-0.78

Table S2. Ad11k $\Delta G^{binding}$ values for CD46(SCR1-2)-Ad11k. Bold faced residues are involved in Coulombic interactions. Letters in parentheses show the protein chain where the mutation occurs. Blue color indicates lysine and arginine mutations, and red color indicates aspartic and glutamic acid mutations.

Mutant	$\Delta G^{binding}$ (kJ/mol)	Mutant	$\Delta G^{binding}$ (kJ/mol)
WT	0	D129A (A)	0.08
		D129A (E)	0.03
H188A (A)	0.04	D155A (A)	1.00
H188A (E)	-0.01	D155A (E)	0.06
H218A (A)	0	D200A (A)	-0.27
H218A (E)	0	D200A (E)	-0.14
		D246A (A)	-0.41
K157A (A)	-0.02	D246A (E)	-0.06
K157A (E)	0.06	D265A (A)	-0.08
K164A (A)	-0.01	D265A (E)	1.39
K164A (E)	0.06	D272A (A)	0.51
K213A (A)	-0.83	D272A (E)	0.18
K213 (E)	0	**D284A (A)**	**3.74**
K219A (A)	-0.03	D284A (E)	-0.25
K219A (E)	0.01	D300A (A)	-0.13
K233A (A)	0.06	**D300A (E)**	**1.46**
K233A (E)	0.14	D324A (A)	0
K251A (A)	0.37	D324A (E)	-0.06
K251A (E)	0	D325A (A)	0.04
		D325A (E)	0.05
R189A (A)	0.39		
R189A (E)	-0.19	E142A (A)	0.03
R208A (A)	1.02	E142A (E)	0.11
R208A (E)	0.10	E152A (A)	0.88
R249A (A)	0.28	E152A (E)	0.12
R249A (E)	0.03	**E196A (A)**	**3.84**
R266A (A)	0.10	E196A (E)	-0.04
**R266A (E)**	**4.28**	E250A (A)	-0.50
**R279A (A)**	**3.15**	E250A (E)	-0.04
R279A (E)	0.07	E252A (A)	-0.23
**R280A (A)**	**3.70**	E252A (E)	-0.01
R280A (E)	0.09	**E285A (A)**	**-4.29**
R291A (A)	-1.12	E285A (E)	-0.19
R291A (E)	0.06	E303A (A)	-0.05
R322A (A)	0.01	E303A (E)	0.96
R322A (E)	0.03	E323A (A)	-0.16
		E323A (E)	-0.09

Table S3. Ad21k $\Delta G^{binding}$ values for CD46(SCR1-2)-Ad21k. Bold faced residues are involved in Coulombic interactions. Letters in parentheses show the protein chain where the mutation occurs. Blue color indicates lysine and arginine mutations, and red color indicates aspartic and glutamic acid mutations.

Mutant	$\Delta G^{binding}$ (kJ/mol)	Mutant	$\Delta G^{binding}$ (kJ/mol)
WT	0	D151A (B)	1.06
		D151A (C)	0.51
H254A (B)	-0.03	D154A (B)	1.23
H254A (C)	0.01	D154A (C)	0.50
		D179A (B)	-0.02
K139A (B)	-0.08	**D179A (C)**	**1.86**
K139A (C)	0	D199A (B)	-0.11
K156A (B)	-0.04	D199A (C)	0.10
K156A (C)	-0.38	D207A (B)	-0.45
K163A (B)	-0.05	D207A (C)	0.25
K163A (C)	-0.04	D248A (B)	-0.01
K188A (B)	0.05	D248A (C)	-0.04
K188A (C)	-0.14	D264A (B)	-0.20
**K212A (B)**	**-1.90**	D264A (C)	-0.81
K212A (C)	-0.30	D321A (B)	0.04
K216A (B)	-0.08	D321A (C)	-0.01
K216A (C)	-0.16	D322A (B)	0.04
K218A (B)	-0.03	D322A (C)	0
K218A (C)	-0.07		
K231A (B)	0.08	**E148A (B)**	**1.62**
K231A (C)	0.01	E148A (C)	0.31
K298A (B)	0.06	**E208A (B)**	**-0.99**
K298A (C)	0.03	E208A (C)	0.18
		E225A (B)	0.04
**R195A (B)**	**-5.30**	E225A (C)	0.03
R195A (C)	-0.22	E250A (B)	0.37
**R247A (B)**	**0.91**	E250A (C)	-0.05
R247A (C)	-0.07	**E292A (B)**	**2.02**
R265A (B)	0.20	E292A (C)	0.16
R265A (C)	1.66	E299A (B)	-0.11
**R279A (B)**	**3.29**	**E299A (C)**	**1.22**
R279A (C)	-0.04	E302A (B)	-0.10
R319A (B)	-0.07	**E302A (C)**	**2.47**
R319A (C)	-0.03	E320A (B)	-0.06
		E320A (C)	-0.01

Figure Captions

Figure S1. Thermodynamic cycle used for the calculation of electrostatic free energies of binding in CD46(SCR1-2)-Ad11k and CD46(SCR1-2)-Ad21k. The top horizontal process represents binding in the reference state (or Coulombic) and the bottom horizontal process represents binding in the solution state. The vertical processes represent solvation processes for the free proteins and the complex. For each state, the dielectric constant of the protein interior ε_p and solvent ε_s, as well as the ionic strength of the solution κ are shown. The desired electrostatic free energy of binding is given by

$$\Delta G^{solution} = \Delta\Delta G^{solvation} + \Delta G^{reference} \quad \text{(S1)}$$

where

$$\begin{aligned} \Delta\Delta G^{solvation} &= \Delta G_{AB}^{solvation} - \Delta G_{A}^{solvation} - \Delta G_{B}^{solvation} \\ \Delta G_{X}^{solvation} &= G_{X}^{solution} - G_{X}^{reference}, \quad X = A, B, AB \end{aligned} \quad \text{(S2)}$$

are calculated using the linearized Poisson-Boltzmann equation of APBS, and

$$\Delta G^{reference} = G_{AB}^{reference} - G_{A}^{reference} - G_{B}^{reference} = \Delta G^{Coulombic} \quad \text{(S3)}$$

is calculated using Coulomb's law with the APBS utility COULOMB. In Eqs. (2) and (3), AB refers to the complexes CD46(SCR1-2)-Ad11k or CD46(SCR1-2)-Ad21k, and A or B refers to CD46(SCR1-2) or Ad11k/Ad21k, respectively, and their mutants.

Figure S2. Electrostatic free energies of binding for Ad11k mutants in CD46(SCR1-2)-Ad11k. Charged residues in CD46(SCR1-2)-Ad11k were systematically mutated into alanine, one at a time as described in text, to generate a family of as many mutants as ionizable residues in the protein. The type of residue, residue number, and chain letter (in parentheses) for each mutant are shown. Negative and positive $\Delta G^{binding}$ values represent increased and decreased thermodynamic stability of mutant complexes relative to WT (parent), respectively. Highlighted residues are involved in specific binding and are grouped based on the distance of their closest Coulombic interaction. All other charged residues have Coulombic interactions more than 8 Å apart. The order of Ad11k mutations in the horizontal axis corresponds to the order in the dendrogram of Supplementary Fig. S6.

Figure S3. Electrostatic free energies of binding for Ad21k mutants in CD46(SCR1-2)-Ad21k. Presentation and notation is as in Supplementary Fig. S2. The order of Ad21k mutants in the horizontal axis corresponds to the order in the dendrogram of Supplementary Fig. S7.

Figure S4. Clustering dendrogram of electrostatic similarity distance (ESD) for CD46(SCR1-2) mutants in the CD46(SCR1-2)-Ad11k complex. Charged residues in CD46(SCR1-2) were systematically mutated into alanine, one at a time as described in text, to generate a family of as many mutants as ionizable residues in the protein. The type of residue, residue number, and chain letter (in parentheses) for each mutant are shown. The horizontal line connecting any two mutants shows the degree of similarity/dissimilarity (ESD) of the spatial distributions of their electrostatic potentials, with zero denoting identical potentials and values greater than zero denoting increasing dissimilarity. The order of mutants in the horizontal axis is as in Fig. 4A of main text. Mutations of positively charged residues (blue) and negatively charged residues (red) residues tend to be clustered together.

Figure S5. Clustering dendrogram of electrostatic similarity distance (ESD) for CD46(SCR1-2) mutants in the CD46(SCR1-2)-Ad21k complex. Presentation and notation is as in Supplementary Fig. S4. The order of mutants in the horizontal axis is as in Fig. 4B of main text.

Figure S6. Clustering dendrogram of electrostatic similarity distance (ESD) for Ad11k mutants in the CD46(SCR1-2)-Ad11 complex. Presentation and notation is as in Supplementary Fig. S4. The order of mutants in the horizontal axis is as in Supplementary Fig. S2.

Figure S7. Clustering dendrogram of electrostatic similarity distance (ESD) for Ad21k mutants in the CD46(SCR1-2)-Ad21 complex. Presentation and notation is as in Supplementary Fig. S4. The order of mutants in the horizontal axis is as in Supplementary Fig. S3.

Figure S8. Decomposition of electrostatic free energies of binding for CD46(SCR1-2) mutants in CD46(SCR1-2)-Ad11k. (A) $\Delta G^{Coulombic}$ according to Supplementary Eq. (S3). (B) $\Delta\Delta G^{solvation}$ according to Supplementary Eq. (S2). (C) $\Delta G^{solution}$ according to Supplementary Eq. (S1). (D) Relative electrostatic free energy of binding of each mutant with regard to that of the parent (marked as WT), $\Delta G^{binding} = \Delta G^{solution}_{mutant} - \Delta G^{solution}_{parent}$, corresponding to Eq. (1) of main text. The graph of the panel (D) is identical to the graph of Fig. 4A of main text, and is included here for comparison and to facilitate the identification of each mutant. Units are in kJ/mol.

Figure S9. Decomposition of electrostatic free energies of binding for CD46(SCR1-2) mutants in CD46(SCR1-2)-Ad21k. The order of panel presentation (A-D) is as in Supplementary Fig. S8. The graph of panel (D) is identical to the graph of Fig. 4B of main text, and is included here to facilitate the comparison across panels and the identification of each mutant. Units are in kJ/mol.

Figure S10. Decomposition of electrostatic free energies of binding for Ad11k mutants in CD46(SCR1-2)-Ad11k. The order of panel presentation (A-D) is as in Supplementary Fig. S8. The graph of the panel (D) is identical to the graph of Supplementary Fig. S2, and is included here to facilitate the comparison across panels and the identification of each mutant. Units are in kJ/mol.

Figure S11. Decomposition of electrostatic free energies of binding for Ad21k mutants in CD46(SCR1-2)-Ad21k. The order of panel presentation (A-D) is as in Supplementary Fig. S8. The graph of panel (D) is identical to the graph of Supplementary Fig. S3, and is included here to facilitate the comparison across panels and the identification of each mutant. Units are in kJ/mol.

Figure S1

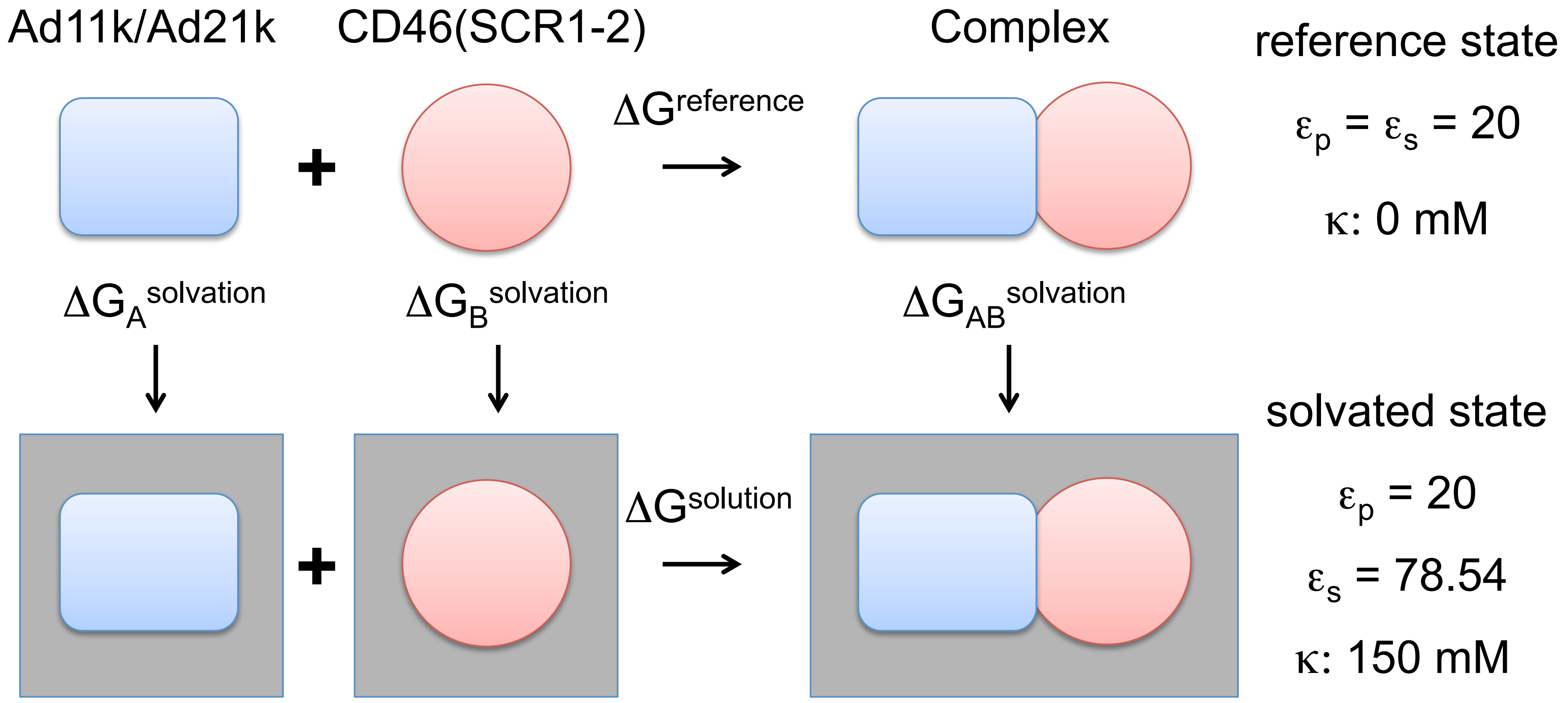

Ad11k/Ad21k
CD46(SCR1-2)
Complex
reference state
$\varepsilon_p = \varepsilon_s = 20$
κ: 0 mM
$\Delta G^{reference}$
$\Delta G_A^{solvation}$
$\Delta G_B^{solvation}$
$\Delta G_{AB}^{solvation}$
solvated state
$\varepsilon_p = 20$
$\varepsilon_s = 78.54$
κ: 150 mM
$\Delta G^{solution}$

Figure S2

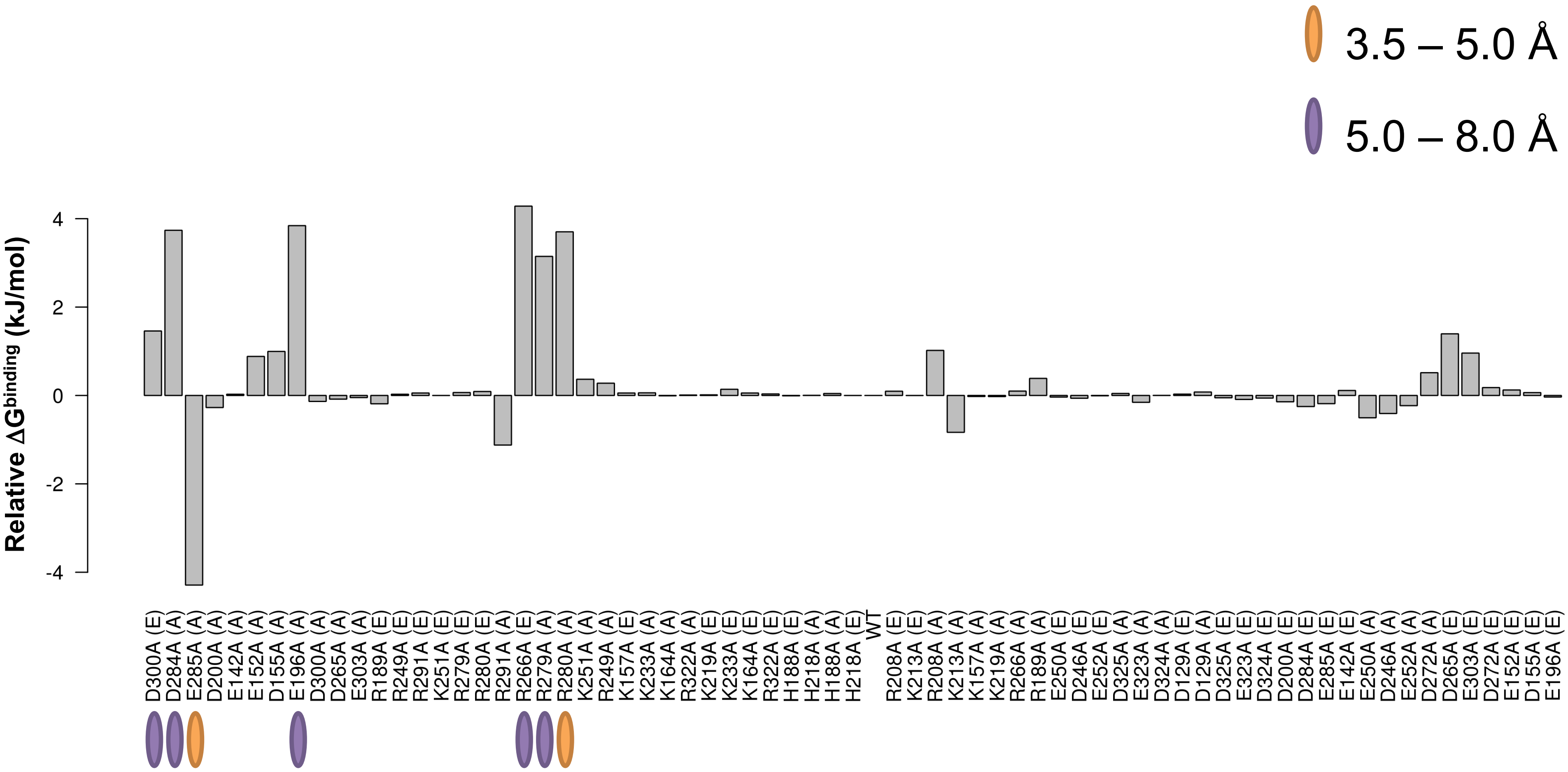

Ad11k Electrostatic Free Energy Graph

Figure S3

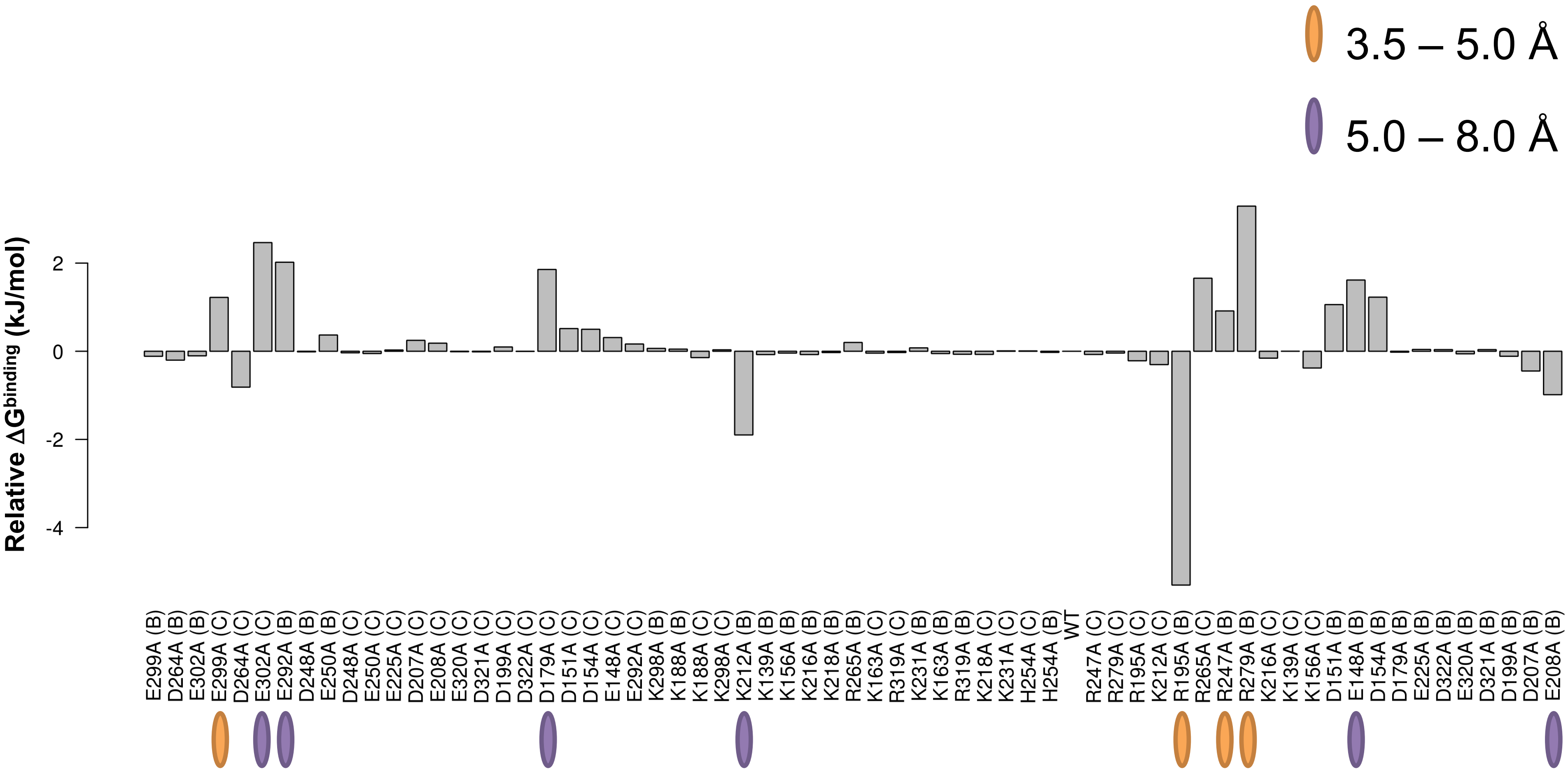

Ad21k Electrostatic Free Energy Graph

Figure S4

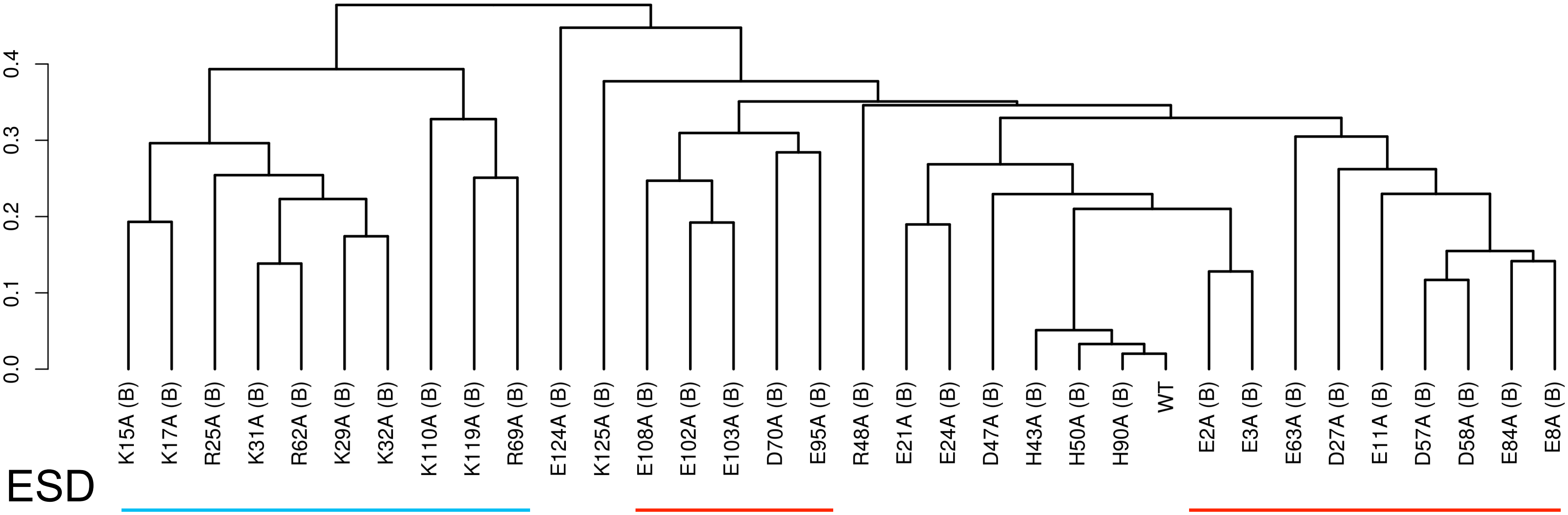

CD46(SCR1-2) Dendrogram (for Ad11k binding)

Figure S5

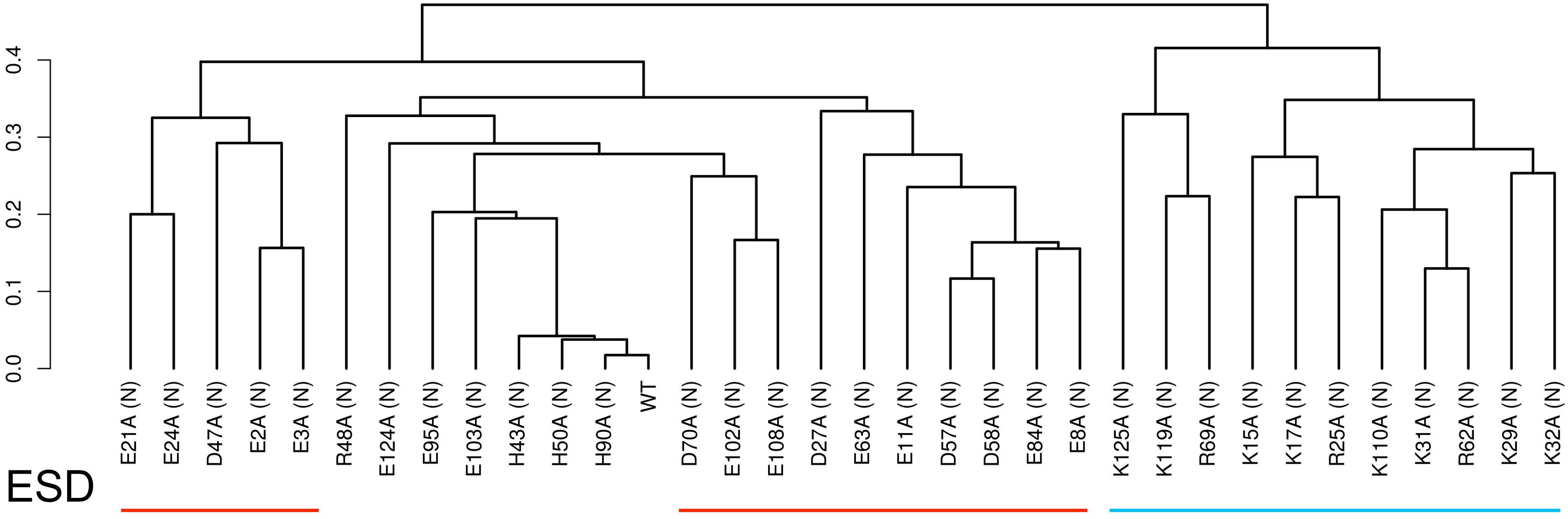

CD46(SCR1-2) Dendrogram (for Ad21k binding)

Figure S6

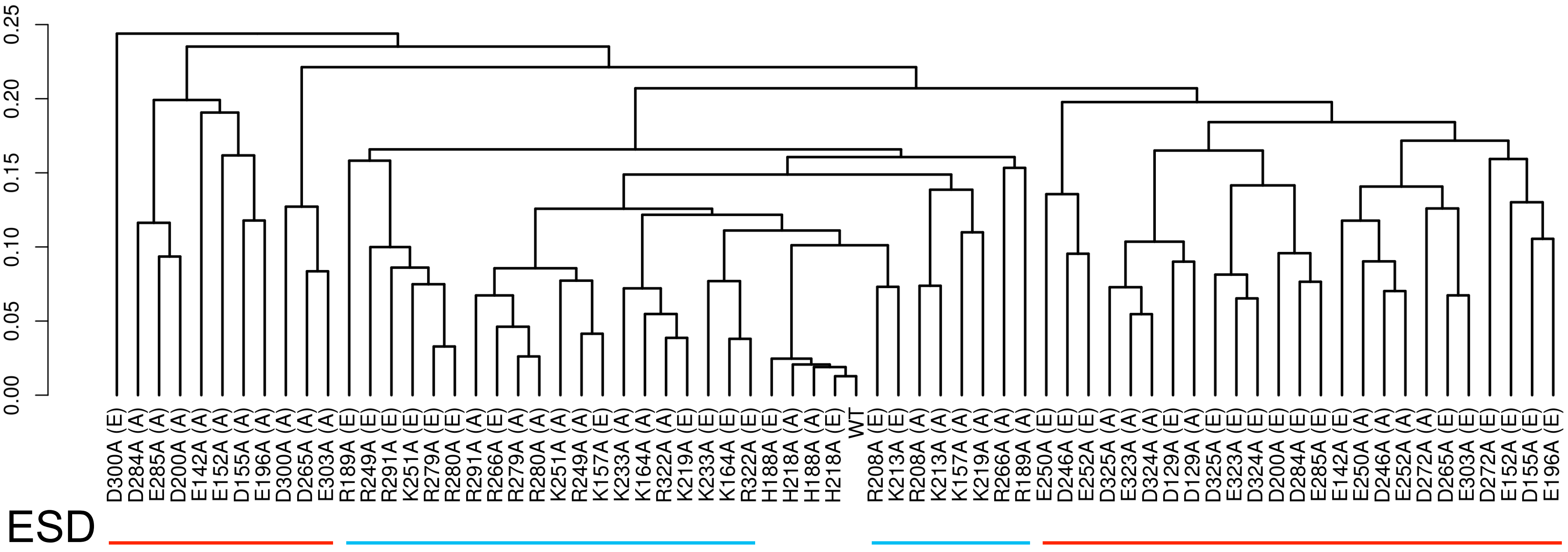

Ad11k Dendrogram

Figure S7

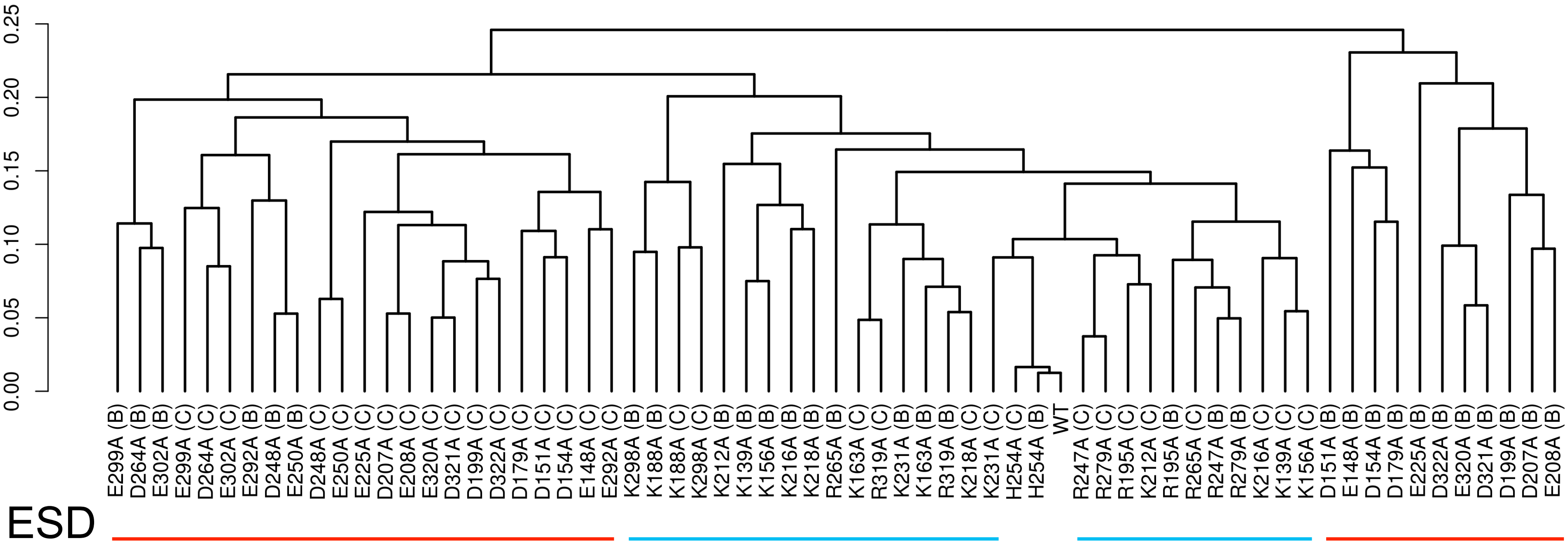

Ad21k Dendrogram

Figure S8

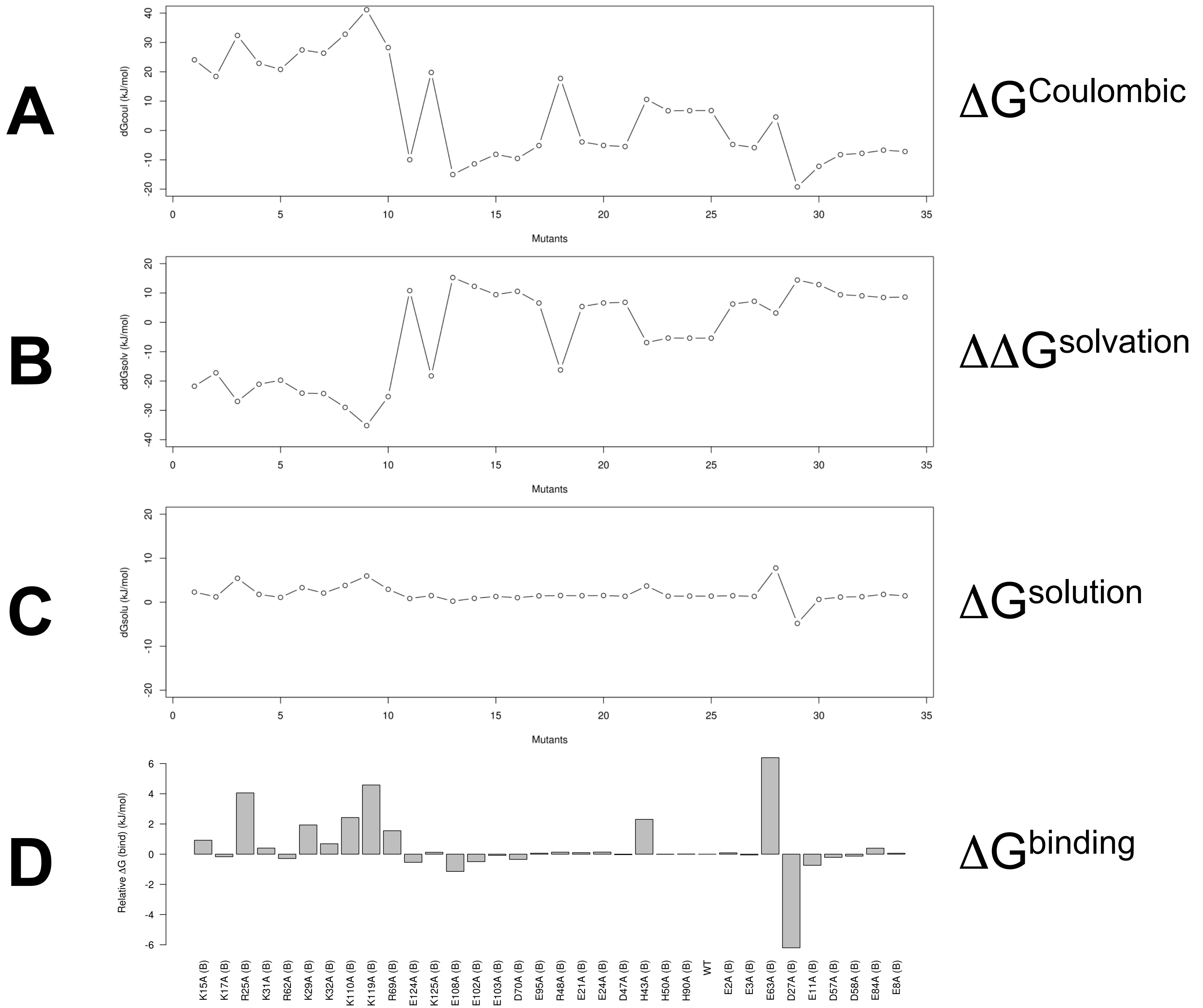

CD46(SCR1-2) Electrostatic Free Energy Decomposition (for Ad11k binding)

Figure S9

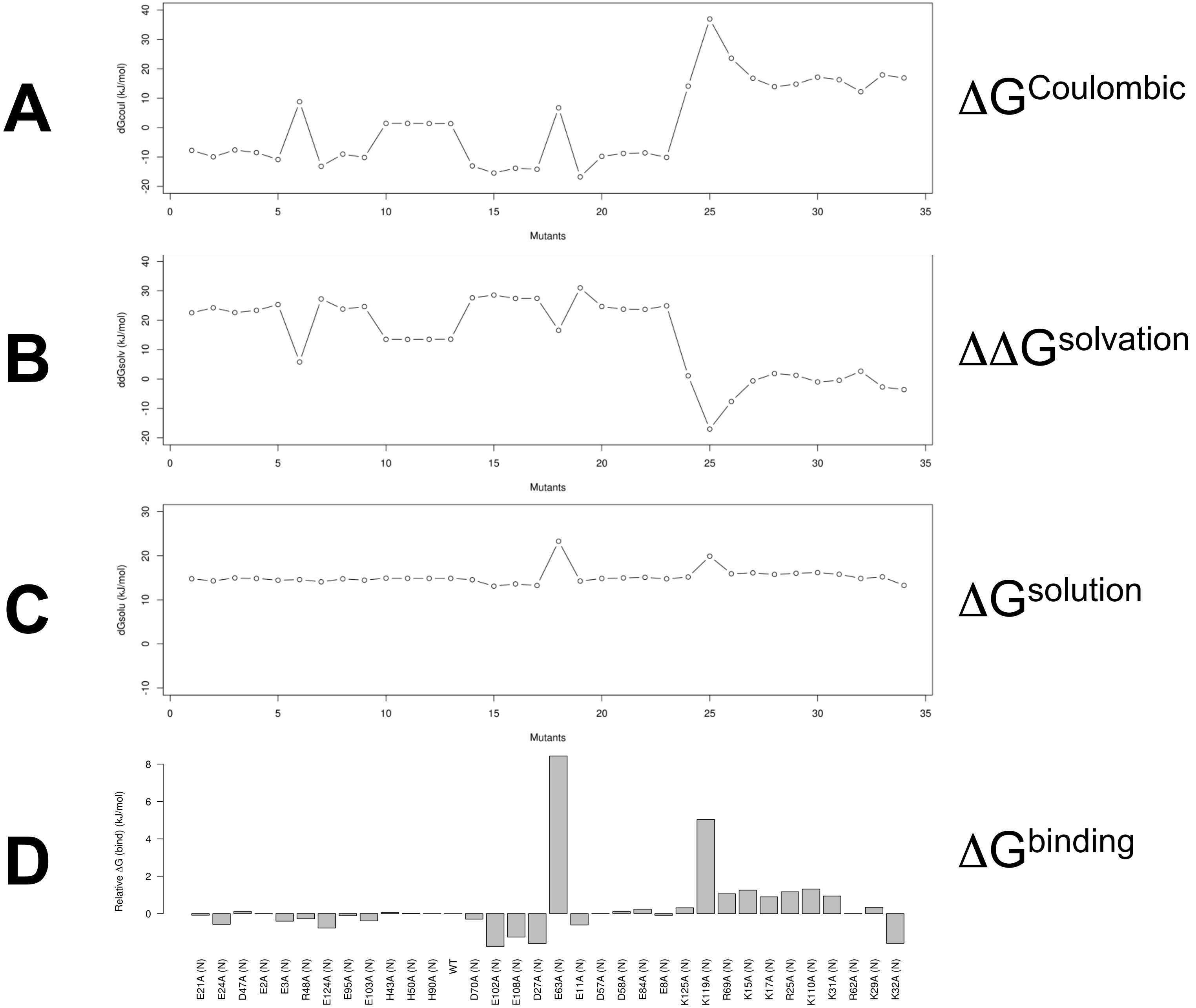

CD46(SCR1-2) Electrostatic Free Energy Decomposition (for Ad21k binding)

Figure S10

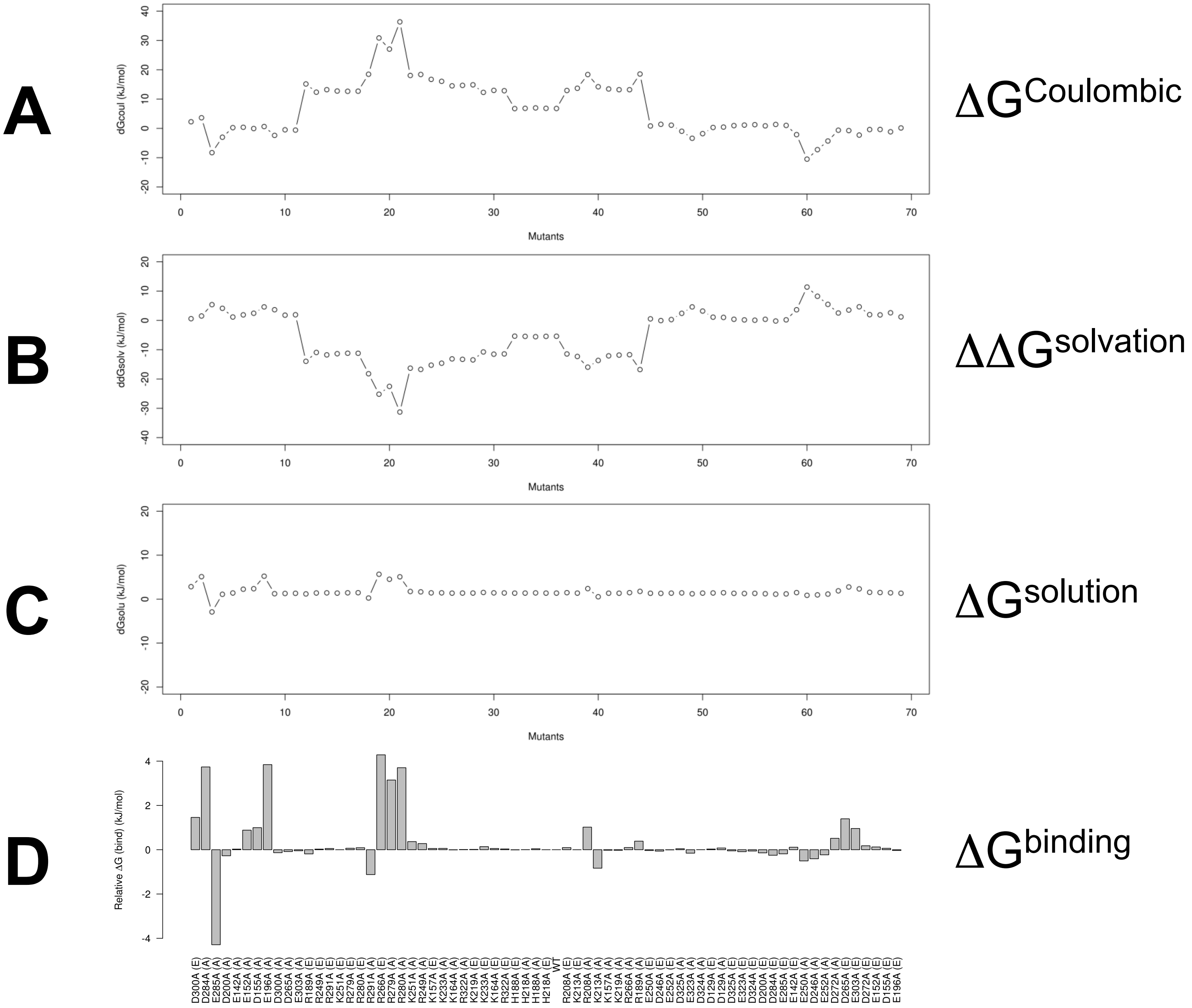

Ad11k Electrostatic Free Energy Decomposition

Figure S11

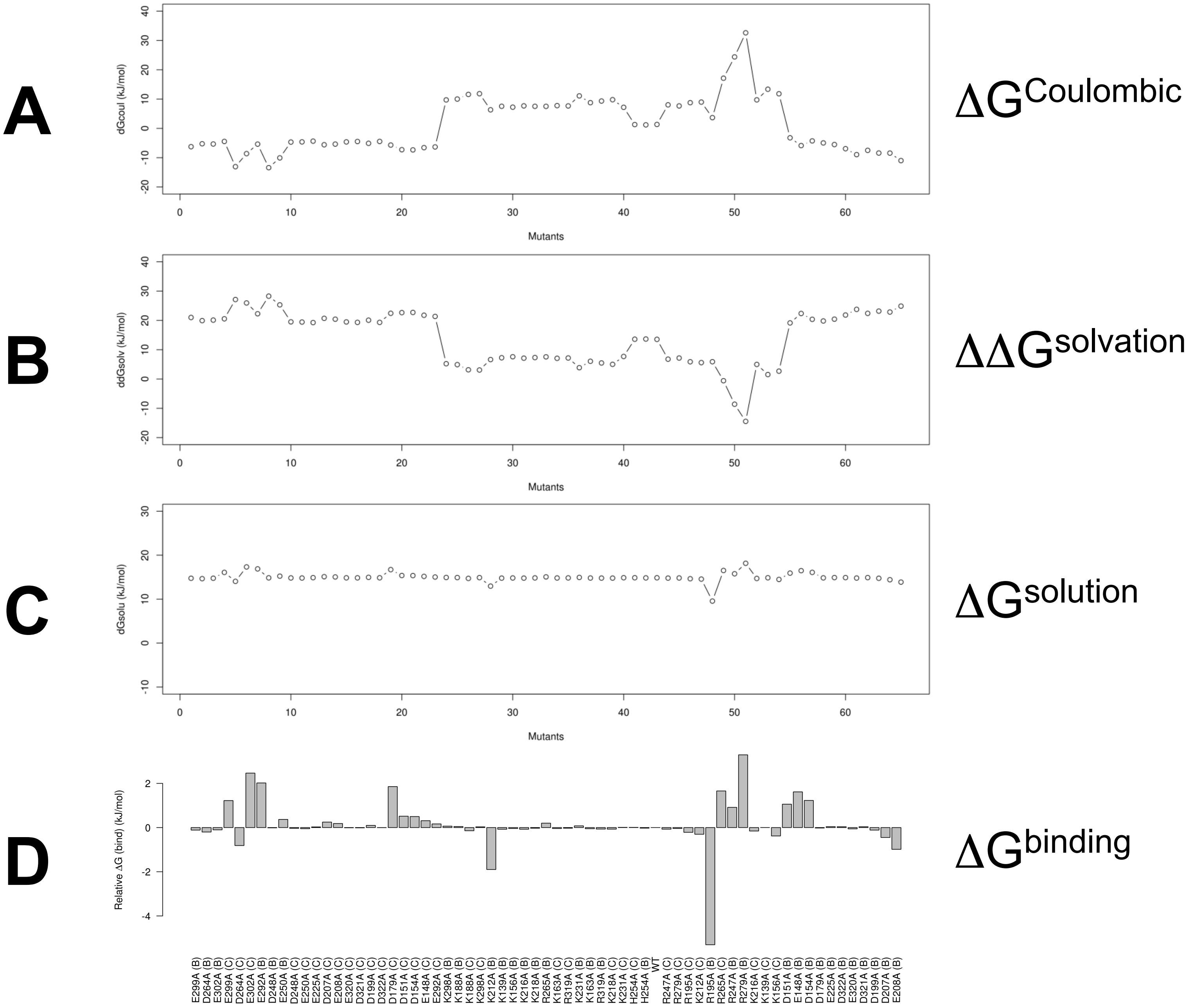

Ad21k Electrostatic Free Energy Decomposition
